# Regulation of Hfq by the RNA CrcZ in *Pseudomonas aeruginosa* Carbon Catabolite Repression

**DOI:** 10.1371/journal.pgen.1004440

**Published:** 2014-06-19

**Authors:** Elisabeth Sonnleitner, Udo Bläsi

**Affiliations:** Department of Microbiology, Immunobiology and Genetics, Max F. Perutz Laboratories, Center of Molecular Biology, University of Vienna, Vienna, Austria; The University of Texas Health Science Center at Houston, United States of America

## Abstract

Carbon Catabolite repression (CCR) allows a fast adaptation of Bacteria to changing nutrient supplies. The *Pseudomonas aeruginosa* (PAO1) catabolite repression control protein (Crc) was deemed to act as a translational regulator, repressing functions involved in uptake and utilization of carbon sources. However, Crc of PAO1 was recently shown to be devoid of RNA binding activity. In this study the RNA chaperone Hfq was identified as the principle post-transcriptional regulator of CCR in PAO1. Hfq is shown to bind to A-rich sequences within the ribosome binding site of the model mRNA *amiE*, and to repress translation *in vitro* and *in vivo*. We further report that Crc plays an unknown ancillary role, as full-fledged repression of *amiE* and other CCR-regulated mRNAs *in vivo* required its presence. Moreover, we show that the regulatory RNA CrcZ, transcription of which is augmented when CCR is alleviated, binds to Hfq with high affinity. This study on CCR in PAO1 revealed a novel concept for Hfq function, wherein the regulatory RNA CrcZ acts as a decoy to abrogate Hfq-mediated translational repression of catabolic genes and thus highlights the central role of RNA based regulation in CCR of PAO1.

## Author Summary

Carbon assimilation in Bacteria is governed by a mechanism known as carbon catabolite repression (CCR). In contrast to several other bacterial clades CCR in *Pseudomonas* species appears to be primarily regulated at the post-transcriptional level. In this study, we have identified the RNA chaperone Hfq as the principle post-transcriptional regulator of CCR in *P. aeruginosa* (PAO1). Hfq is shown to act as a translational regulator and to prevent ribosome loading through binding to A-rich sequences within the ribosome binding site of mRNAs, which encode enzymes involved in carbon utilization. It has been previously shown that the synthesis of the RNA CrcZ is augmented in the presence of non-preferred carbon sources. Here, we show that the CrcZ RNA binds to and sequesters Hfq, which in turn abrogates Hfq-mediated translational repression of mRNAs, the encoded functions of which are required for the breakdown of non-preferred carbon sources. This novel mechanistic twist on Hfq function not only highlights the central role of RNA based regulation in CCR of PAO1 but also broadens the view of Hfq-mediated post-transcriptional mechanisms.

## Introduction

The opportunistic human pathogen *Pseudomonas aeruginosa* causes acute as well as chronic infections in immunocompromised individuals. Moreover, airway epithelia of patients suffering from cystic fibrosis are frequently colonized by the pathogen [1]. *P. aeruginosa* is a metabolically versatile organism with the ability to utilize numerous carbon sources, which allows the bacterium to thrive in different environments such as soil, marine habitats as well as on/in different organisms [2].

In Bacteria, the uptake and utilization of carbon compounds is controlled in a hierarchical manner by a mechanism known as *c*arbon *c*atabolite *r*epression (CCR). Generally speaking, CCR prevents the utilization of less preferred carbon sources until the preferred one is consumed. In *Escherichia coli* CCR prevents the expression of catabolic genes, the transcription of which requires the transcriptional activator CRP (*c*yclic AMP *r*eceptor *p*rotein) in conjunction with cAMP, whereas in *Bacillus subtilis* CCR is mediated by the transcriptional repressor CcpA (*c*atabolite *c*ontrol *p*rotein *A*). In both organisms CCR is regulated by a signal transduction pathway inherent to the phosphoenolpyruvate-carbohydrate phosphotransferase system [3].

In most studied *Pseudomonas* spp. the presence of organic acids (for example succinate) results in CCR, which leads to repression of catabolic genes required for the consumption of other carbon sources. During CCR catabolic genes were deemed to be down-regulated by the translational repressor Crc (*c*atabolite *r*epression *c*ontrol protein) [4]. It has been suggested that Crc binds to CA-rich motifs within or adjacent to *r*ibosome *b*inding *s*ites (RBS) of multiple target mRNAs, and thereby prevents their translation [5]–[7]. Upon relief of CCR, the regulatory RNAs, CrcZ in PAO1 [7], CrcZ/CrcY in *P. putida* [8] and CrcZ/CrcX in *P. syringae* [9] were proposed to bind to and to counteract Crc by trapping the protein. This hypothesis was in line with the observation that the CrcZ levels increase in the presence of poor carbon sources, and that they are reduced in the presence of a preferred carbon source [7]. However, our recent structural and biochemical studies challenged the role of Crc as a direct translational repressor of genes governed by CCR in PAO1. Recombinant Crc purified to homogeneity did neither bind to *amiE* mRNA, encoding aliphatic amidase, nor to CrcZ RNA [10], [11]. Rather, the previously reported RNA binding activity of His-tagged Crc purified by nickel affinity chromatography [6], [7] was attributed to a contamination of the Crc-His preparations with the RNA chaperone Hfq [10], [11].

In *Enterobacteriaceae* Hfq is pivotal for riboregulation [12], [13], which results on the one hand from binding to and protection of sRNAs from nucleolytic decay [14], and on the other hand from accelerating base-pairing between sRNAs and their target mRNAs [15]–[17]. *E. coli* Hfq hexamers have dedicated RNA binding sites, preferably binding uridine-rich stretches of sRNAs around the central pore of the proximal surface [18], [19] and A-rich sequences on the distal surface [20]. In addition, the lateral surface of the hexamer can as well contribute to sRNA binding [21]. The dedicated sRNA and mRNA binding surfaces on either site of the Hfq-hexamer may serve to transiently increase the local concentration of two RNA substrates. Moreover, the inherent capacity of Hfq to induce conformational changes in RNAs together with the observed structural flexibility of RNA ligands bound to Hfq could stochastically facilitate base-pairing [22], [23].

Although many sRNA candidates have been identified in PAO1 [24]–[27], the function of only a few has been revealed. The sRNAs PhrS [28] and PrrF [29] have been shown and inferred, respectively, to act by base-pairing with target mRNAs, whereas the protein binding RNAs RsmY and RsmZ are known to antagonize the function of the translational regulator RsmA [30]. PAO1 Hfq was shown to stabilize the protein binding RNA RsmY [31], [32] and to affect expression of some sRNAs including PhrS [25]. In PAO1, Hfq acts as a pleiotropic regulator, impacting on growth, virulence, motility, and quorum sensing [31], [33]. A transcriptome analysis of a PAO1*hfq*- strain revealed that ∼15% of all genes were de-regulated. These included a number of genes encoding proteins involved in carbon compound catabolism, which were up-regulated in the absence of Hfq [31].

Here, we studied the impact of Hfq on CCR in PAO1. *In vivo* and *in vitro* studies revealed that Hfq acts as a translational repressor of several catabolic genes. Moreover, we present evidence that the regulatory RNA CrcZ binds to and sequesters Hfq, which in turn results in translation of Hfq-regulated mRNAs. Hence, this study revealed a novel mechanistic twist on post-transcriptional regulation by Hfq and highlights its role in regulating the central metabolism in *P. aeruginosa*.

## Results

### Hfq represses catabolic genes at the post-transcriptional level

Several observations suggested a link between Hfq and CCR in PAO1. A comparative transcriptome analysis of a PAO1 wt and a PAO1*hfq-* strain disclosed transcripts encoding functions related to carbon compound and amino-acid catabolism that were up-regulated in the absence of Hfq ([31]; Table S1). Many of these transcripts comprise A-rich stretches (Table S1) within or adjacent to the RBS, which could serve as recognition motifs for the distal poly-A binding site of Hfq [20], [34]. Mutations within these A-rich stretches in certain mRNAs abrogated CCR [7], [35].

Crc was deemed to act as a translational regulator in PAO1 CCR [5]–[7]. However, Crc was recently shown to be deficient in binding to CrcZ and to the CCR regulated *amiE* mRNA [10]. In fact, these studies identified Hfq as a contaminant RNA binding activity in Crc preparations [10], [11]. This observation prompted us to test (i) whether Hfq serves as the principle post-transcriptional regulator of CCR in PAO1, and (ii) whether the regulatory RNA CrcZ, displaying several A-rich motifs [7], might abrogate Hfq-mediated regulation by sequestering Hfq.

We first revisited post-transcriptional regulation of *amiE* mRNA, encoding an aliphatic amidase. Strain PAO1(pTC*amiE*) and strain PAO1(pME9655) harboring a plasmid borne transcriptional *amiE-lacZ* and a translational *amiE::lacZ* fusion, respectively, were grown in BSM medium either in the presence of succinate/acetamide (CCR) or mannitol/acetamide (no CCR). Acetamide was added to induce transcription of the chimeric *amiE* genes, i.e. to mimic CCR. As shown in Figure S1A (left panel), the β-galactosidase activities conferred by the transcriptional fusion was comparable in either medium, i.e. in the presence and absence of CCR. However, when compared with growth in the presence of mannitol/acetamide (no CCR), *amiE::lacZ* translation was repressed in strain PAO1(pME9655) (Figure S1A; right panel) during cultivation in the presence of succinate/acetamide, i.e. when CCR was in place.

We next tested whether *amiE* mRNA and CrcZ RNA associate with Hfq upon induction of *amiE* transcription during CCR. Strain PAO1*hfq*- harboring plasmid pMMB*hfq*_Flag_ (encodes flag-tagged Hfq) and the control plasmid pMMB67HE, respectively, was grown to an OD_600_ of 1.5 in BSM medium supplemented with succinate. In addition, acetamide was added to induce transcription of the *amiE* gene, i.e. to mimic CCR. Then, cell lysates were prepared and Hfq-associated RNAs were co-immunoprecipitated (CoIP) with Hfq-specific antibodies. As revealed by RT-PCR both, *amiE* and CrcZ, were found in complex with Hfq (Figure S2). In contrast RsmZ, which does not bind to Hfq [7], could not be detected among the RNAs that co-immunoprecipitated with Hfq (Figure S2). Taken together, these initial studies validated *amiE* as a model mRNA to scrutinize the hypothesized role of Hfq in CCR.

**Figure 1:**
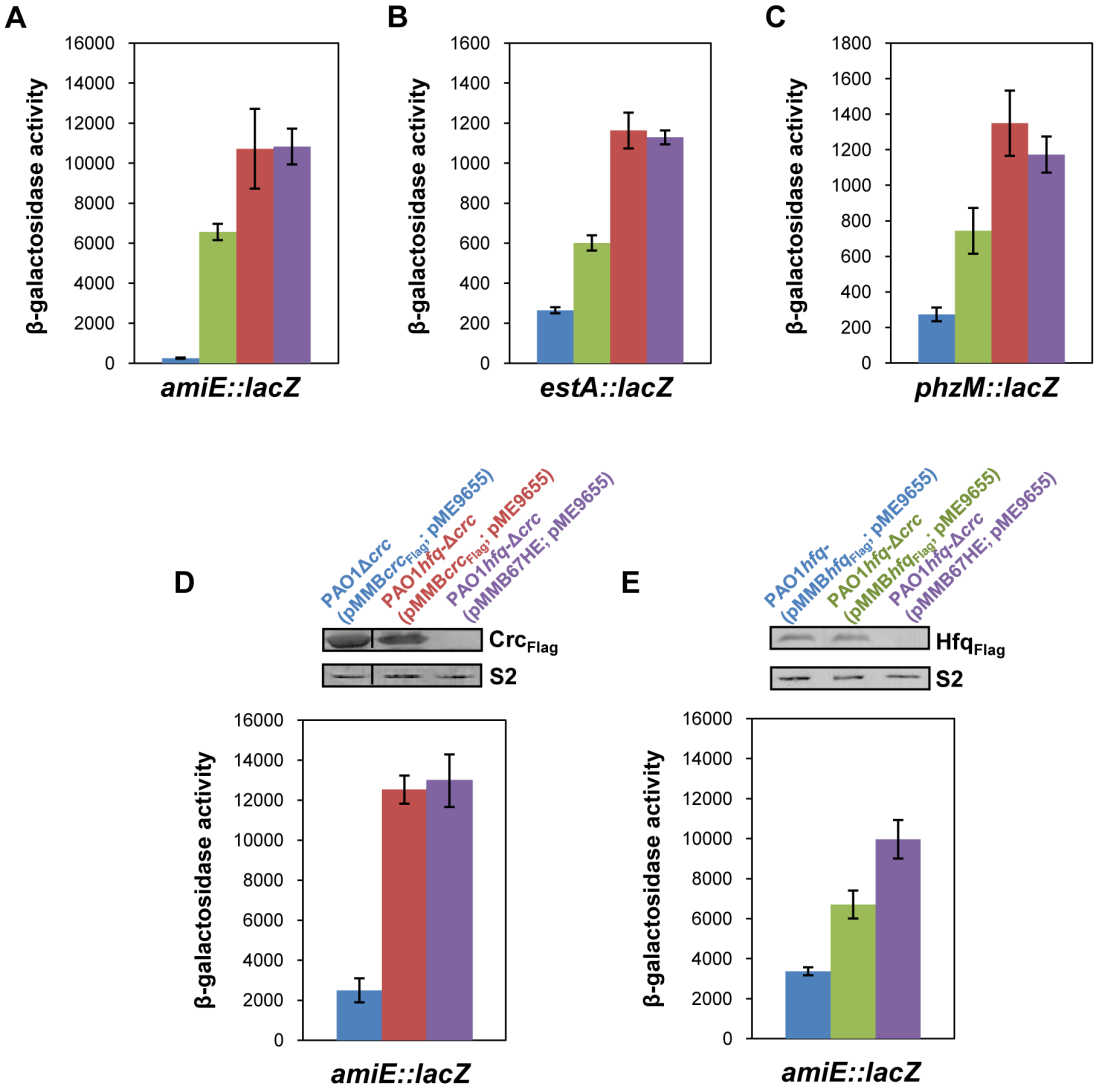
Repression of catabolic genes by Hfq. (**A–C**) The strains were grown to an OD_600_ of 2.0 in BSM medium supplemented with (**A**) 40 mM succinate and 40 mM acetamide (to establish CCR and to induce *amiE::lacZ* transcription) or (**B**, **C**) only with 40 mM succinate (CCR). Then, the cells were harvested and the β-galactosidase activities were determined. The bars depict β-galactosidase values conferred by the translational *amiE::lacZ* fusion encoded by plasmid pME9655 (**A**), the *estA::lacZ* fusion encoded by plasmid pTL*estA* (**B**) and the *phzM*::*lacZ* fusion encoded by plasmid pME10011 (**C**) in strains PAO1 (blue bar), PAO1Δ*crc* (green bar), PAO1*hfq*- (red bar) and PAO1*hfq*-Δ*crc* (purple bar), respectively. The error bars represent standard deviations from three independent experiments. (**D, E**) The strains were grown in BSM medium supplemented with succinate and acetamide. (**D**) At an OD_600_ of 1.0 IPTG (1 mM final concentration) was added to strains PAO1Δ*crc*(pMMB*crc*_Flag_; pME9655) (blue bar), PAO1*hfq*-Δ*crc*(pMMB*crc*_Flag_; pME9655) (red bar) and PAO1*hfq*-Δ*crc*(pMMB67HE; pME9655) (purple bar), respectively. The bars represent the β-galactosidase values conferred by the plasmid pME9655 encoded translational *amiE::lacZ* fusion 3 h after induction of the plasmid borne *crc*_Flag_ gene. The error bars represent standard deviations from three independent experiments. The corresponding Crc_Flag_ levels were determined by western-blot analysis using anti-Flag antibodies in strains PAO1Δ*crc*(pMMB*crc*_Flag_; pME9655), PAO1*hfq*-Δ*crc*(pMMB*crc*_Flag_; pME9655) and PAO1*hfq*-Δ*crc*(pMMB67HE; pME9655). Immunodetection of ribosomal protein S2 (loading control) was performed as described in Materials and Methods. (**E**) At an OD_600_ of 1.0 IPTG (1 mM final concentration) was added to strains PAO1*hfq*-(pMMB*hfq*_Flag_; pME9655) (blue bar), PAO1*hfq*-Δ*crc*(pMMB*hfq*_Flag_; pME9655) (green bar) and PAO1*hfq*-Δ*crc*(pMMB67HE; pME9655) (purple bar), respectively. The bars represent the β-galactosidase values conferred by the plasmid pME9655 encoded translational *amiE::lacZ* fusion 3 h after *hfq*_Flag_ induction. The error bars represent standard deviations from three independent experiments. The corresponding Hfq_Flag_ levels were determined by western-blot analysis using anti-Flag antibodies in strains PAO1*hfq*-(pMMB*hfq*_Flag_; pME9655), PAO1*hfq*-Δ*crc*(pMMB*hfq*_Flag_; pME9655) and PAO1*hfq*-Δ*crc*(pMMB67HE; pME9655) (top panel). Immunodetection of ribosomal protein S2 (loading control) was performed as described in Materials and Methods.

To obtain first hints whether Hfq is involved in post-transcriptional regulation of *amiE* during CCR, the strains PAO1, PAO1*hfq*-, PAO1Δ*crc* and PAO1*hfq*-Δ*crc* were transformed with plasmid pTC*amiE* harboring the transcriptional *amiE-lacZ* fusion and with plasmid pME9655 harboring the translational *amiE::lacZ* fusion, respectively. The strains were grown in BSM medium in the presence of succinate to establish CCR, and in the presence of acetamide to induce *amiE-lacZ*/*amiE::lacZ* transcription. The β-galactosidase activity conferred by the transcriptional *amiE-lacZ* fusion was comparable in the presence and absence of Hfq and/or Crc in either strain (Figure S1B). In contrast, the β-galactosidase activity conferred by the translational *amiE::lacZ* fusion differed in strains PAO1, PAO1*hfq*-, PAO1Δ*crc* and PAO1*hfq*-Δ*crc* when grown in BSM medium containing succinate and acetamide (CCR). In contrast to PAO1, *amiE::lacZ* translation was greatly increased in the PAO1*hfq*- strain and in the PAO1*hfq*-Δ*crc* double mutant, respectively (Figure 1A). The absence of Crc resulted as well in marked *amiE::lacZ* translation, albeit at a lower level when compared with the *hfq-* mutant or the *hfq-*Δ*crc* double mutant, suggesting that Hfq exerts a more pronounced negative effect on *amiE* translation during CCR than Crc. To verify these experiments, we tested whether Hfq likewise affects translation of the *estA* and the *phzM* genes, which are also known to be regulated by CCR [7], [35], [36]. The impact of Hfq was again monitored using translational *lacZ* reporter gene fusions. The results obtained mirrored those obtained with the *amiE::lacZ* reporter gene. Their translation was repressed during growth of PAO1 in BSM medium containing succinate, whereas in the absence of Hfq, Crc or both, the synthesis of the encoded fusion proteins increased (Figures 1B and C). In contrast, the expression of the heterologous *lacZ* gene (variation control), was comparable in strains PAO1, PAO1*hfq*-, PAO1Δ*crc* and PAO1*hfq*-Δ*crc* (Figure S1C). Taken together, these initial studies supported our hypothesis that Hfq is involved in post-transcriptional regulation of CCR regulated genes.

### Hfq acts as the principle post-transcriptional regulator and Crc has an auxiliary function in CCR

As shown in Figure 1A–C, both, Crc and Hfq, were required for full repression of all three CCR regulated genes. Although translation occurred in the absence of Crc, a pronounced increase in translation only required the absence of Hfq, *i.e.* the observed de-repression was comparable in the *hfq-* strain and in the *hfq-*Δ*crc* double mutant. We interpreted this as showing that Hfq acts as the principal translational repressor, whereas Crc seemed to act as an auxiliary factor, somehow amplifying the negative regulation exerted by Hfq. To further test this hypothesis, *amiE::lacZ* translation was monitored in the PAO1*hfq*-Δ*crc* double mutant complemented with a plasmid borne *crc*_Flag_ and *hfq*_Flag_ gene, respectively. In consideration that *crc* could impinge on *hfq* expression and *vice versa*, the plasmid pMMB*crc*_Flag_ and pMMB*hfq*_Flag_ borne *crc*_Flag_ and *hfq*_Flag_ genes, respectively, were equipped with the same expression signals, i.e. their expression was controlled by the P*_tac_* promoter and identical translation initiation signals. The different strains used in this experiment were grown in BSM medium supplemented with succinate and acetamide (CCR). At an OD_600_ of 1.0, IPTG was added to induce ectopic expression of the *crc*_Flag_ and *hfq*_Flag_ genes, respectively. Three hours thereafter, *amiE::lacZ* translation was monitored by determination of the β-galactosidase activities and the Crc-Flag and Hfq-Flag levels were determined by quantitative western-blot analysis.

As shown in Figure 1D (blue bar), under these conditions *amiE::lacZ* translation was repressed in strain PAO1Δ*crc*(pMMB*crc*_Flag_; pME9655). In contrast, translation of the *amiE::lacZ* fusion gene was de-repressed in the absence of Hfq in the double mutant PAO1*hfq-*Δ*crc*(pMMB*crc*_Flag_; pME9655) (Figure 1D; red bar). Moreover, when compared with the control strain PAO1*hfq*-Δ*crc*(pMMB67HE; pME9655) (Figure 1D, purple bar), ectopic expression of *crc*_Flag_ in strain PAO1*hfq-*Δ*crc*(pMMB*crc*_Flag_; pME9655) did not affect *amiE::lacZ* translation in the absence of Hfq.

Next, *amiE::lacZ* translation was monitored in strains PAO1*hfq-*(pMMB*hfq*_Flag_, pME9655) and PAO1*hfq*-Δ*crc*(pMMB*hfq*_Flag_; pME9655) after growth in BSM succinate/acetamide medium (CCR) and 3 h after ectopic expression of the *hfq*_Flag_ gene. The absence of Crc in the double mutant strain PAO1*hfq*-Δ*crc*(pMMB*hfq*_Flag_; pME9655) (Figure 1D; green bar) resulted in an increased de-repression of *amiE::lacZ* translation when compared with strain PAO1*hfq-*(pMMB*hfq*_Flag_;, pME9655) (Figure 1D; blue bar) despite comparable levels of Hfq_Flag_ in both strains. Taken together, these experiments showed that Crc only impacts on *amiE::lacZ* translation in the presence of Hfq. Hence, they corroborate the idea that Crc does not act *per se* as a translational regulator but functions as an ancillary factor in Hfq-mediated repression of target genes.

### Hfq binds to the translation initiation region of *amiE* mRNA and prevents *in vitro* ribosome binding and translation

**Figure 2:**
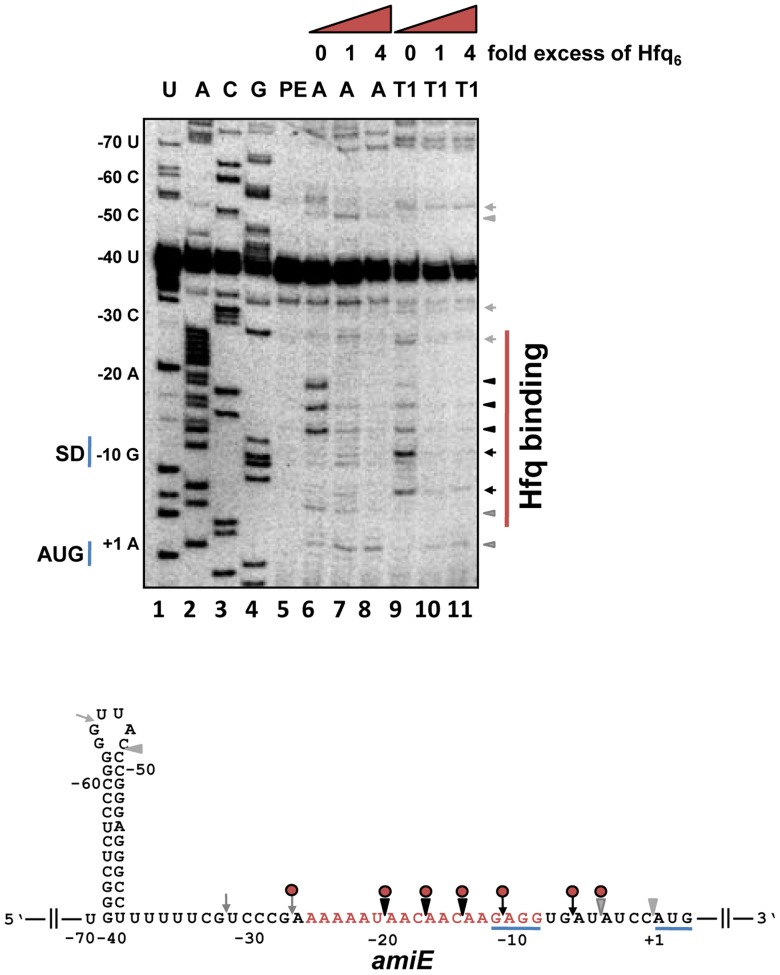
Hfq binding to the RBS of *amiE* mRNA revealed by enzymatic probing. Lanes 1–4: sequencing reactions. Lane 5, primer extension (PE). Lanes 6–8, RNase A (A) cleavage in the absence and in the presence of increasing amounts of Hfq-hexamer (Hfq_6_). Lanes 9–11, RNase T1 (T1) cleavage in the absence and in the presence of increasing amounts of Hfq_6_. The nucleotides indicated on the left of the autoradiograph and in the RNA sequence (below) are numbered with regard to the A (+1) of the *amiE* start codon. Arrowheads and arrows denote RNase A and RNase T1 cleavage, respectively. Black and grey symbols indicate strong and weak cleavage, respectively. Red circles indicate protection from RNase cleavage by Hfq. The region protected by Hfq is indicated by a red bar on the right of the autoradiograph. Only the relevant part of the autoradiograph is shown. The A-rich stretch from nt position −26 to −8, which can be potentially accommodated in the six distal binding pockets of Hfq, are indicated in red in the *amiE* RNA sequence. The Shine and Dalgarno sequence and the start codon of *amiE* are underlined.

Next, we tested whether Hfq directly represses translation of *amiE* mRNA by binding to the translation initiation region (TIR). First, a filter binding assay was performed with purified PAO1 Hfq and an *amiE* mRNA fragment encompassing nucleotides (nt) from position −134 to +20 with regard to the A (+1) of the start codon. This experiment revealed that Hfq binds to *amiE*_−134–+20_ with a K_d_ of ∼67.0±1.4 nM (Figure S3). Next, the Hfq binding site(s) were mapped on *amiE* RNA. Enzymatic probing was performed with riboendonucleases T1 (G-specific cleavage) and A (C/U-specific cleavage) in the absence and presence of Hfq. As shown in Figure 2, Hfq protected the segment of *amiE* RNA extending from G_−28_ to U_−5_. This region includes the Shine and Dalgarno sequence (SD) sequence of *amiE* mRNA. Thus, Hfq binding to this region would readily explain the observed translational repression of *amiE::lacZ* mRNA (Figure 1A, D, E).

To further test whether Hfq acts as a translational repressor of *amiE* mRNA, the PURExpress system was employed. The *in vitro* translation system was programmed with *amiE*_Flag_ mRNA, encoding a Flag-tagged amidase. As shown in Figure S4, translation of *amiE*_Flag_ mRNA was already impeded at a 1∶1 molar ratio of Hfq to mRNA. As the *in vitro* system is reconstituted from purified components of the translation machinery of *E. coli*, no additional PAO1 component was apparently required for repression.

**Figure 3:**
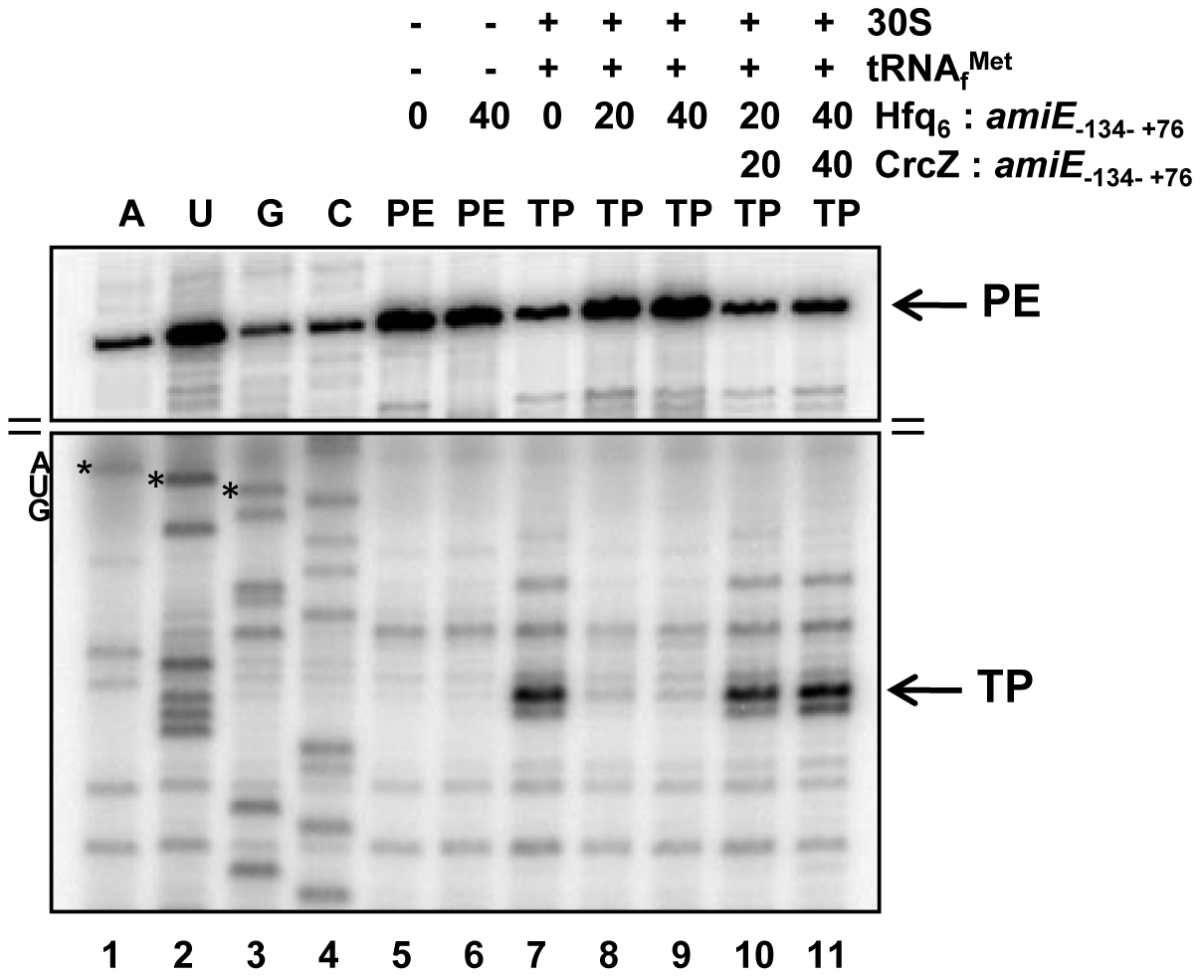
Hfq inhibits translation initiation complex formation on *amiE* mRNA. Lanes 1–4, sequencing reactions. Lane 5, primer extension (PE) in the absence of 30S subunits, tRNA^fMet^, and Hfq. Lane 6, primer extension in the presence of Hfq. Lane 7, toeprinting with 30S subunits and tRNA^fMet^ in the absence of Hfq. Lanes 8 and 9, toeprinting with 30S subunits and tRNA^fMet^ in the presence of Hfq. Lanes 10 and 11, toeprinting with 30S subunits and tRNA^fMet^ in the presence of CrcZ and Hfq. CrcZ and/or Hfq were added concomitantly with *amiE* mRNA, 30S subunits and tRNA^fMet^. The molar ratios of Hfq-hexamer (Hfq_6_) and CrcZ RNA to *amiE* mRNA are indicated on top. Hfq and CrcZ were added in equimolar ratios. The arrows denote the toeprint signal (TP) at position +17 with regard to the A (+1) of the start codon and the primer extension signals (PE). Only the relevant parts of the autoradiogram are shown. The position of the AUG start codon is indicated.

To further demonstrate that Hfq directly interferes with ribosome binding, a toeprinting assay was performed with *amiE*_−134–+76_ mRNA in the presence and absence of Hfq. Briefly, in the presence of tRNA^fMet^, 30S ribosomes form a stable ternary complex at the RBS of mRNAs, which can be visualized by inhibition of cDNA synthesis primed downstream of the start codon [37]. A toeprint signal usually occurs at position +15 to +17 with regard to the A (+1) of the start codon. As shown in Figure 3, lane 7, a toeprint signal at the *amiE* RBS was observed in the absence of Hfq, whereas the addition of Hfq to *amiE*_−134–+76_ mRNA inhibited ternary complex formation (Figure 3, lanes 8 and 9). In contrast, the presence of Hfq alone did not result in a stop signal (Figure 3, lane 6), which is in accordance with earlier observations that translational repressors do not always provide a roadblock for cDNA synthesis by reverse transcriptase under these conditions [38]. Taken together, these *in vitro* studies strongly supported the idea that Hfq acts as a translational repressor that prevents ribosome loading on *amiE* mRNA.

### The distal site of Hfq is required for regulation of *amiE* mRNA

**Figure 4:**
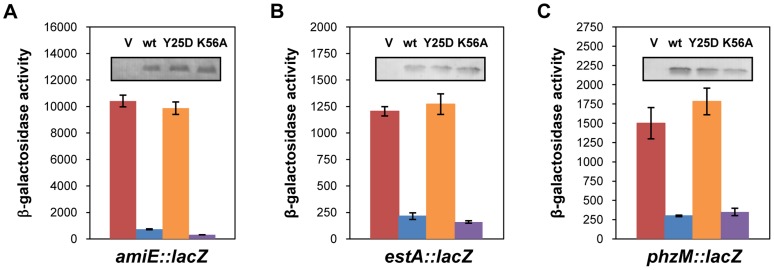
The distal poly-A binding site of Hfq is required for *amiE* repression. The cultures were grown to an OD_600_ of 2.0 in BSM medium supplemented with (**A**) 40 mM succinate and 40 mM acetamide or (**B**, **C**) only with 40 mM succinate. The β-galactosidase activity conferred by the translational *amiE::lacZ* fusion encoded by plasmid pME9655 (**A**), the *estA::lacZ* fusion encoded by plasmid pTL*estA* (**B**) and the *phzM*::*lacZ* fusion encoded by plasmid pME10011 (**C**) was determined in strain PAO1*hfq*-(pME9655) harboring the control plasmid pME4510 (red bar), plasmid pME4510*hfq*_Flag_ (blue bar), plasmid pME4510*hfq*_Y25DFlag_ (orange bar) and pME4510*hfq*_K56AFlag_ (purple bar), respectively. Inset: the levels of Hfq_Flag_, Hfq_Y25DFlag_ and Hfq_K56AFlag_ were determined by western-blot analysis using anti-Flag antibodies. The error bars represent standard deviations from three independent experiments.

Link et al. [20] reported a crystal structure of *E. coli* Hfq in complex with poly(A_15_), wherein the poly(A) tract is bound to the distal face using tripartite binding motifs. They consist of an adenosine specific site (A- site), a purine nucleotide selectivity site (R-site) and a sequence-non-discriminating E-site. The amino-acids involved in building the A-R-N motifs are fully conserved in the Hfq protein of PAO1 [34]. As the hexamer Hfq could accommodate the entire A-rich stretch from nt −26 to −8 of *amiE* mRNA (Figure 2) in the six binding pockets, we next tested whether the distal binding site of Hfq is required and sufficient for translational repression of *amiE* mRNA. We therefore engineered the PAO1 *hfq* variants *hfq*_Y25DFlag_ and *hfq*_K56AFlag_ as the corresponding *E. coli* mutant proteins were shown to be deficient in binding to polyA- and polyU-tracts [18], respectively. In contrast to the PAO1 *hfq_Flag_* gene and the PAO1 *hfq_K56AFlag_* allele, ectopic expression of the *hfq_Y25DFlag_* allele did not result in repression of *amiE::lacZ* translation, albeit all three proteins, Hfq_Flag_, Hfq_Y25DFlag_ and Hfq_K56AFlag_, were present at comparable levels (Figure 4A). Basically the same results were obtained when translation of the *estA::lacZ* (Figure 4B) and *phzM::lacZ* (Figure 4C) fusion genes was monitored in the presence of Hfq_Flag_, Hfq_Y25DFlag_ and Hfq_K56AFlag_, respectively. Hence, translational repression of these reporter genes apparently required an intact distal polyA binding site of Hfq.

To verify these *in vivo* data, the PAO1 Hfq variants Hfq_Y25D_ and Hfq_K56A_ were purified, and binding to *amiE*_−134–+20_ mRNA was assessed using electrophoretic mobility shift assays (EMSA). As shown in Figure S5A, while PAO1 Hfq and Hfq_K56A_ bound to the mRNA fragment, the Hfq_Y25D_ protein failed to bind. With increasing concentrations of either Hfq or Hfq_K56A_ two shifted bands were observed (Figure S5A), suggesting that Hfq binds at two sites of the *amiE*_−134–+20_ fragment. This observation can be explained by the Hfq binding site mapped between nucleotides G_−28_ to U_−5_ (Figure 2) and by a probable second binding site, comprising an A-rich region (nucleotides −92 to −71), which was inferred from an RNomics approach after CoIP with Hfq-specific antibodies [25]. Taken the in *vivo* and *in vitro* studies together, these experiments strongly suggested that Hfq binds with its distal face to the RBS of *amiE*, and by inference most likely also to *estA and phzM* mRNA.

To further corroborate the idea that Hfq directly represses *amiE* translation the experiment was also performed in the heterologous *E. coli hfq-* strain JW4130. The strain was transformed with the control plasmid pME4510 and derivatives thereof harboring the PAO1 *hfq_Flag_* gene, the PAO1 *hfq*_Y25DFlag_ allele and the PAO1 *hfq*_K56AFlag_ allele, respectively. In addition, these strains were transformed with plasmid pME9658, wherein the *amiL* terminator preceding the *amiE* gene was deleted. As shown in Figure S5B, the experimental results paralleled that performed in PAO1. The translation of the *amiE::lacZ* gene was repressed in the presence of Hfq_Flag_ and Hfq_K56AFlag_, whereas the reporter gene was translated in the presence of Hfq_Y25DFlag_. Thus, the presence of Hfq was apparently necessary and sufficient for repression of *amiE* translation in *E. coli*.

### The regulatory RNA CrcZ titrates Hfq *in vitro*

The regulatory RNA CrcZ is present at lower levels during CCR, when compared to conditions when CCR is not in place, e.g. in BSM medium containing mannitol as the sole carbon source [7]. The 407 nt long CrcZ RNA contains six A-rich stretches (Figure S6A) to which Hfq can potentially bind with its distal surface, i.e. with the same binding surface as it binds to the RBS of *amiE* (Figure S5A). Binding of Hfq to the first 151 nt of CrcZ, containing 3 A-rich stretches (Figure S6A) was confirmed using EMSA assays (Figure S6B). Three shifted bands could be discerned (Figure S6B, lane 2), which would be consistent with one Hfq-hexamer binding to either A-rich stretch. As anticipated, the PAO1 Hfq_Y25D_ mutant protein was defective in binding to CrcZ_151_, whereas the Hfq_K56A_ variant bound to the CrcZ fragment like native Hfq. Thus, CrcZ binds to the distal face of Hfq like *amiE* mRNA, and therefore has the potential to titrate Hfq, which in turn would explain the observed increase in translation of *amiE::lacZ* mRNA in BSM mannitol medium, i.e. in the absence of CCR (see Figure S1A).

**Figure 5:**
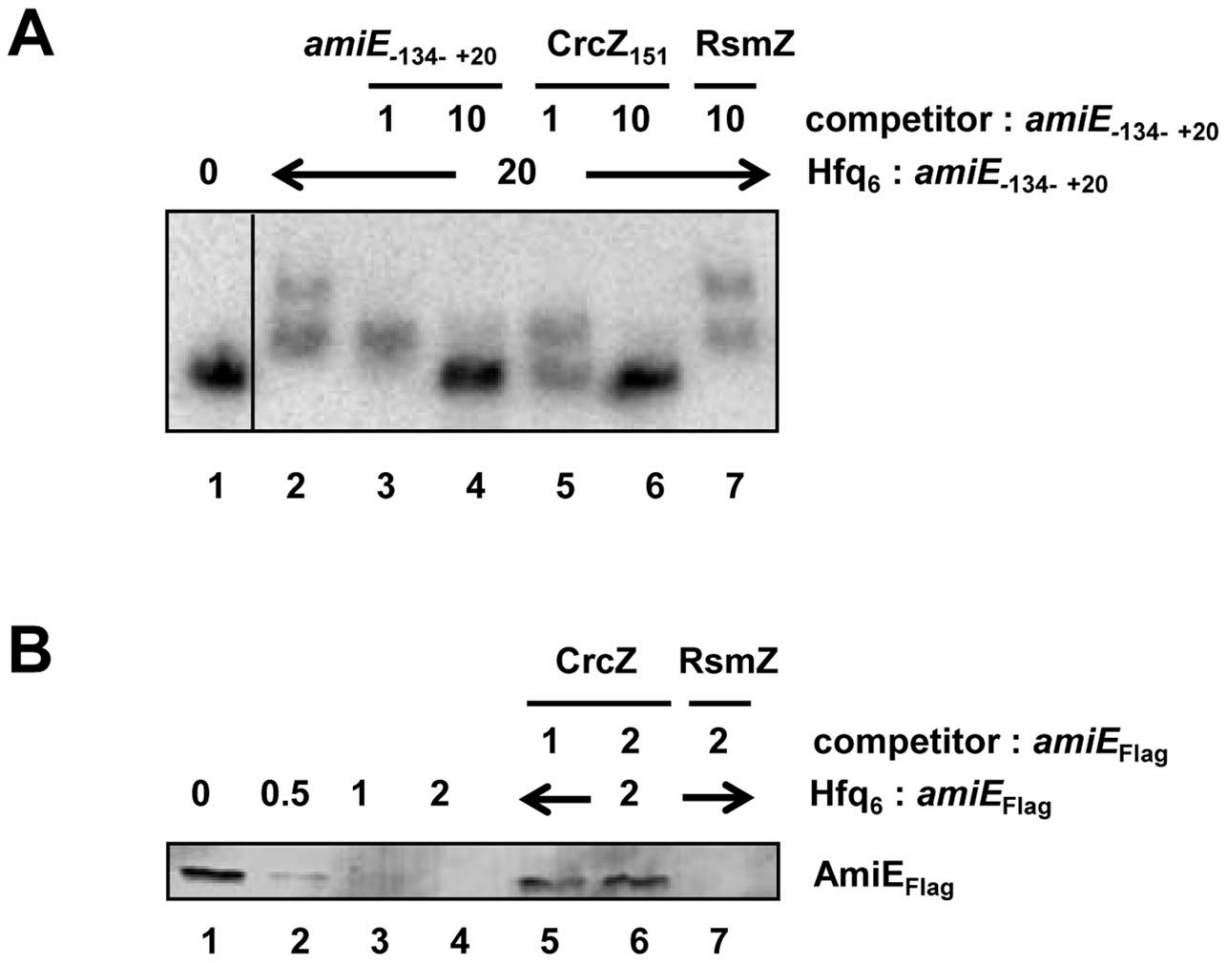
CrcZ competes with *amiE* for Hfq. (**A**) Electrophoretic mobility shift assay with 10 nM radioactively labeled *amiE*_−134–+20_ mRNA in the presence of Hfq protein and unlabelled specific or non-specific competitor RNAs. Lane 1, no protein was added to labeled *amiE*_−134–+20_ mRNA. Lane 2–7, Hfq-hexamer was added in 20-fold molar excess over labeled *amiE*_−134–+20_ RNA. Lanes 3 and 4, 5 and 6, and 7, unlabeled *amiE*_−134–+20_ RNA, unlabelled CrcZ_151_ RNA and unlabelled RsmZ RNA were added, respectively. The molar ratios of the competitor RNAs to *amiE*_−134–+20_ mRNA are shown on top. (**B**) Repression of *amiE*_Flag_ mRNA translation is relieved in the presence of CrcZ. Lane 1, *in vitro* translation of *amiE*_Flag_ mRNA. Lanes 2–4, inhibition of *amiE*_Flag_ mRNA translation in the presence of increasing amounts of Hfq. The molar ratios of Hfq-hexamer to *amiE*_Flag_ mRNA are denoted on top. Lanes 5–7, Hfq was added in 2-fold molar excess over *amiE*_Flag_ mRNA. CrcZ was concomitantly added in equimolar concentration (lane 5) or in two-fold molar excess (lane 6) over Hfq. Lane 7, RsmZ was added in 2-fold molar excess over *amiE*_Flag_ mRNA.

In the next set of experiments we therefore asked whether CrcZ can abrogate Hfq-mediated translational repression *in vitro*. First, a mobility shift assay was performed with radioactively labeled *amiE*_−134–+20_ RNA in the presence of unlabelled specific CrcZ_151_ and non-specific RsmZ competitor RNAs, respectively. As shown in Figure 5A, CrcZ_151_ competed with *amiE*_−134–+20_ for binding to Hfq (Figure 5A, lanes 5 and 6), whereas the addition of RsmZ RNA, which does not bind to Hfq [7], did not result in a downshift of labeled *amiE*_−134–+20_ RNA (Figure 5A, lane 7). When compared with the addition of unlabelled *amiE*_−134–+20_ RNA, the addition of an equimolar concentration of CrcZ_151_ resulted already in a significant downshift, i.e. loss of Hfq binding to *amiE*_−134–+20_. Hence, CrcZ_151_ acted as a better competitor for Hfq than *amiE*_−134–+20_, which is anticipated as the used CrcZ_151_ has three Hfq binding sites, whereas the *amiE* fragment has only two.

Next, *amiE_Flag_* mRNA was translated *in vitro* in the presence of Hfq as well as in the presence of Hfq and CrcZ mRNA. As shown before (Figure S4), Hfq inhibited translation of *amiE*_Flag_, (Figure 5B, lanes 2–4). In contrast, the mRNA was translated in the presence of CrcZ (Figure 5B, lanes 5 and 6), whereas the Hfq-mediated translational repression was not relieved in the presence of the non-specific competitor RNA RsmZ (Figure 5B, lane 7).

Similarly, the addition of CrcZ RNA to the Hfq-*amiE* complex, under conditions that inhibited translation initiation complex formation, resulted in reappearance of a toeprint signal (Figure 3, lanes 10 and 11). Thus, CrcZ counteracted the function of Hfq. Taken together, these *in vitro* experiments indicated that CrcZ can titrate Hfq, and that it can prevent it from binding to the *amiE* RBS.

### CrcZ-mediated regulation of catabolic genes *in vivo* depends on Hfq

**Figure 6:**
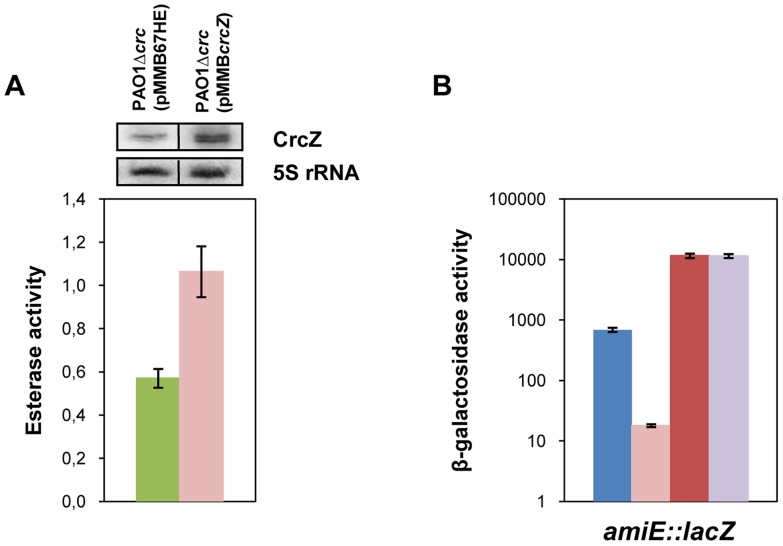
CrcZ relieves repression by Hfq *in vivo*. (**A**) The esterase activity was measured as change in A_410_ min^−1^ per OD_600_ in strain PAO1Δ*crc* harboring either the control plasmid pMMB67HE (green bar) or the *crcZ* encoding plasmid pMMB*crcZ* (pink bar). The strains were grown to an OD_600_ of 1 in BSM medium supplemented with 40 mM succinate. Then, *crcZ* expression was induced by addition of IPTG and samples were withdrawn 60 minutes thereafter. The error bars represent standard deviations from three independent experiments. The CrcZ levels (top panel) were determined by Northern-blot analysis. 5S rRNA served as a loading control. (**B**) The strains were grown to an OD_600_ of 2.0 in BSM medium supplemented with 40 mM succinate and 40 mM acetamide. Then, the cells were harvested and the β-galactosidase activities were determined. The bars depict the β-galactosidase values conferred by the translational *amiE::lacZ* fusion encoded by plasmid pME9655 in strain PAO1 (blue bar), PAO1Δ*crcZ* (pink bar), PAO1*hfq*- (red bar) and PAO1*hfq*-Δ*crcZ* (purple bar). The error bars represent standard deviations from three independent experiments.

In our model titration of Hfq by CrcZ would result from increased CrcZ levels [7] in the presence of non-preferred carbon sources, which in turn would lead to de-repression of Hfq-regulated genes. Hence, it would be anticipated that ectopic over-expression of CrcZ would cause de-repression of Hfq-regulated genes even during CCR, i.e. under conditions when the CrcZ levels are low [7]. With this line of reasoning, we next asked whether ectopic expression of *crcZ* during CCR results in increased expression of the *estA* gene, which was assessed by monitoring the esterase activity. To avoid any interference of Crc, the experiments were performed in a PAO1Δ*crc* strain. The control strain PAO1Δ*crc*(pMMB67HE) and the *crcZ* over-expressing strain PAO1Δ*crc*(pMMB*crcZ*) were grown in BSM medium supplemented with 40 mM succinate to an OD_600_ of 1. Then, *crcZ* expression was induced and samples were taken 60 minutes thereafter. Esterase was produced even without induction of *crcZ*, which can be reconciled with the absence of Crc (see Figure 1B) and by endogenous background expression of *crcZ* in strain PAO1Δ*crc*(pMMB67HE) (Figure 6A, upper panel). Nevertheless, ectopic over-expression of *crcZ* (Figure 6A, upper panel) resulted in elevated activities of esterase (Figure 6A), reflecting de-repression of *estA* translation.

Similarly, over-expression of *crcZ* in PAO1 resulted in de-repression of *amiE::lacZ* translation during CCR in BSM succinate/acetamide medium (Figure S7A), which is in agreement with our model wherein CrcZ titrates Hfq. In contrast to strain PAO1(pMMB*crcZ*;pME9655) (Figure S7A), ectopic expression of *crcZ* did not affect translation of *amiE::lacZ* in the *hfq-* strain PAO1*hfq-*(pMMB*crcZ*;pME9655) (Figure S7B), clearly showing that CrcZ-mediated regulation requires Hfq.

The model would further specify that a deletion of the *crcZ* gene should increase repression of *amiE::lacZ* during CCR. As shown in Figure 6B, when compared with strain PAO1(pME9655), *amiE::lacZ* translation was even further repressed in strain PAO1Δ*crcZ* (pME9655) when grown in BSM medium supplemented with succinate and acetamide (CCR).

Moreover, *amiE::lacZ* translation during CCR was indistinguishable in the absence of Hfq in strain PAO1*hfq-*(pME9655) and in the double mutant PAO1*hfq-*Δ*crcZ*(pME9655). Taken together, these experiments showed that the *hfq* deletion is epistatic to *crcZ*, in other words that CrcZ exerts regulation on *amiE::lacZ* only in the presence of Hfq. Hence, these *in vivo* experiments lend further support to the hypothesis that CrcZ titrates Hfq, and thereby abrogates Hfq-mediated translational repression.

## Discussion

### Target genes of Hfq in PAO1

In this study, we provided evidence that Hfq represses three CCR regulated genes, *amiE*, *estA* and *phzM*. Given that Hfq blocked translation initiation of *amiE* mRNA by binding to A-rich stretches encompassing the RBS, we revisited catabolic and transport genes that were found to be up-regulated in a comparative transcriptome analysis of a PAO1 and a PAO*hfq*- mutant [31]. Out of 126 putative target mRNAs, 72 transcripts contained A-rich stretches in the TIR (Table S1). Out of the latter, 28 transcripts contain several single non-consecutive A-R-N motifs, whereas 44 transcripts contained consecutive (A-R-N)_n_ repeats with n≥3 either within or in close proximity to the RBS (Table S1), indicating that Hfq could interfere with ribosome binding. Although it remains to be tested for either candidate transcript it is tempting to speculate that Hfq is involved in translational regulation of several genes controlled by CCR. In addition, it is conceivable that several CCR controlled genes, which do not contain A-rich regions in the TIR [39], are indirectly controlled by Hfq.

### Hfq in CCR of *Pseudomonas aeruginosa*: regulating a few at a time

**Figure 7:**
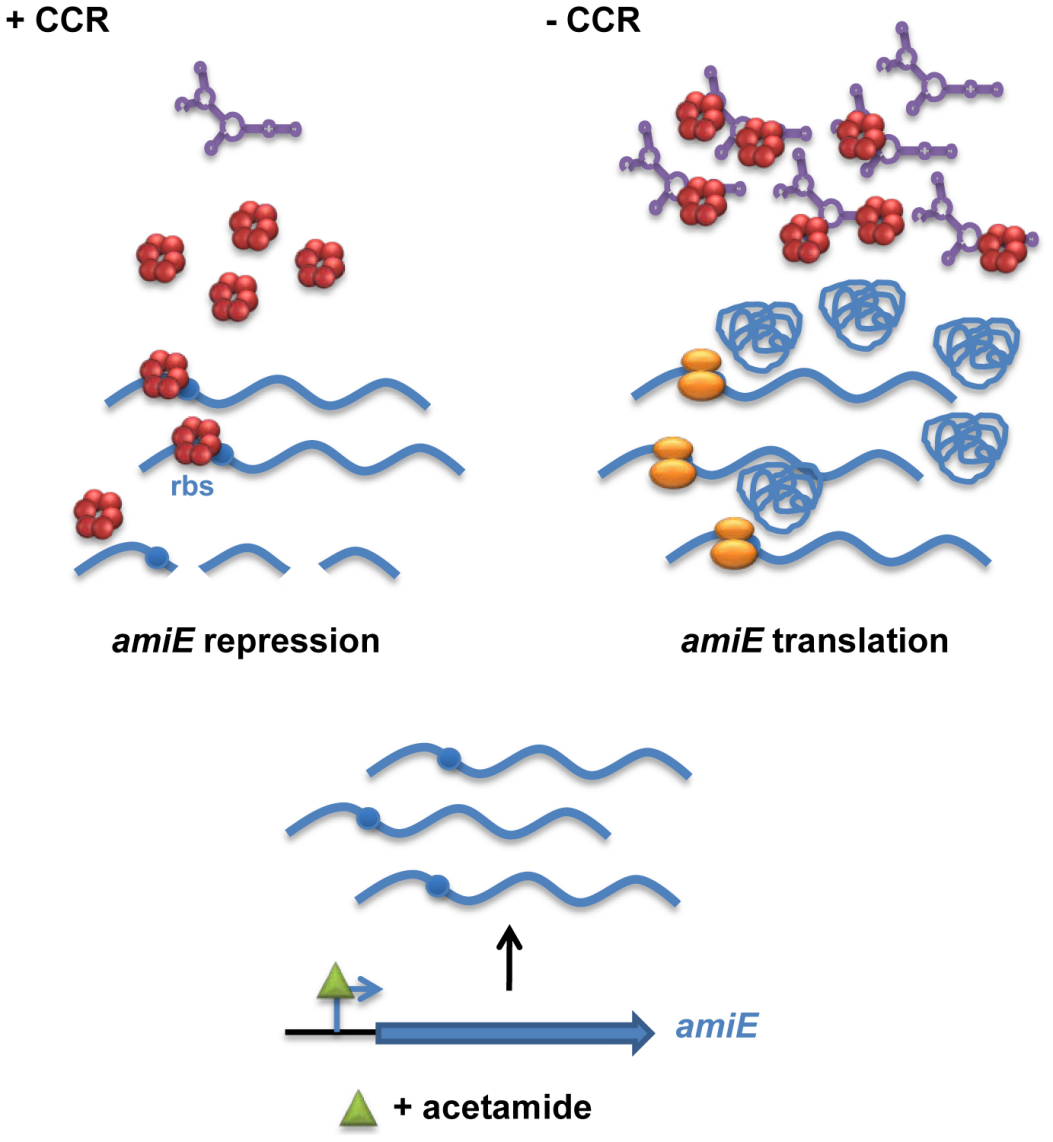
Simplified model for CCR in *Pseudomonas aeruginosa* orchestrated by Hfq and CrcZ. During CCR (left), for example during growth in the presence of succinate, the regulatory RNA CrcZ (in purple) is poorly transcribed. Upon catabolite-dependent induction of catabolic genes, for example induction of *amiE* transcription by acetamide, Hfq (red hexamer) represses translation of the corresponding transcripts, which in turn results in their degradation. When CCR is alleviated (right) the abundance of CrcZ increases. CrcZ binds to and titrates Hfq, which permits translation of catabolic genes, for example *amiE*.

Based on our *in vivo* and *in vitro* studies with the *amiE* model mRNA, we suggest that Hfq acts as a translational repressor of transcripts subjected to CCR in PAO1 (Figure 7). As there appear to be several Hfq regulated genes (Table S1) and our preliminary results show that Hfq levels are more or less constant during growth in different carbon sources (E. Sonnleitner, unpublished), the question arises how Hfq can regulate numerous mRNAs. Many if not all catabolic genes including the corresponding transporter genes are primarily regulated at the transcriptional level [4], [40], and their transcription usually requires the presence of the respective catabolite. Therefore, it is rather unlikely that many of the putative target genes (Table S1) are concomitantly induced at the same time. Thus, in the presence of a preferred carbon source, e.g. succinate, only a few other catabolites may induce concomitant transcription of the corresponding catabolic genes. Moreover, translational repression most likely leads to degradation of mRNAs [41], [42] encoding catabolic enzymes other than those required for the breakdown of succinate, and thus to recycling of Hfq. Therefore, CCR control may not require vast amounts of Hfq. This hypothesis is supported by the experiment shown in Figure S8. When compared with the absence of CCR (presence of acetamide only) or with the PAO1*hfq-* strain, *amiE* mRNA was faster degraded in PAO1 during CCR in the presence of succinate and acetamide. In addition, we have estimated the number of Hfq hexamers/cell in BSM- succinate medium with 2160+/−56 per PAO1 cell (Figure S9), which might suffice to silence “a few catabolic transcripts” that are induced during CCR (Figure 7).

In the presence of non-preferred carbon sources, the two component system CbrAB is activated; phosphorylated CbrB binds to the RpoN-dependent promoter of *crcZ* and stimulates CrcZ synthesis [7], [43]. Our data (Figures 5 and 6) suggest that CrcZ then binds to and titrates Hfq by virtue of its A-rich binding motifs. This in turn would allow translation of transcripts that are induced by available catabolites, and thus synthesis of the cognate degradative enzymes (for example aliphatic amidase) (Figure 7). For the following reasons we favor the hypothesis that no other sRNAs are required for regulation of the catabolic genes examined in this study. Most *E. coli* sRNAs bind to the proximal site of Hfq, and binding is usually abrogated in the Hfq_K56A_ mutant protein [13]. However, in contrast to the Hfq_Y25D_ variant, the Hfq_K56A_ mutant protein was still capable to repress the fusion genes governed by CCR in PAO1 (Figure 4) and in *E. coli* (Figure S5B). It seems therefore reasonable to suggest that regulatory sRNAs other than CrcZ are not involved in Hfq-mediated regulation of these catabolic genes.

### A novel concept for Hfq function: from mediating riboregulation to being regulated

Although binding of *E. coli* Hfq to A-rich stretches in mRNA has been demonstrated in several model systems (see below), Hfq seems to work predominantly in conjunction with sRNAs [44]–[46]. Hfq-mRNA binding may recruit the sRNA to the target mRNA and stimulate sRNA-mRNA pairing. In this scenario, the sRNA competes with initiating 30S ribosomes, whereas Hfq has a rather indirect function.

Some deviations from the canonical model of Hfq assisted and sRNA-mediated regulation of target mRNAs have been reported. In *E. coli* evidence has been provided that Hfq acts as an autogenous repressor on its own mRNA. As for PAO1 *amiE* mRNA (Figure 2), *E. coli* Hfq was shown to bind to an A-rich sequence encompassing the SD sequence of *hfq* mRNA [47]. Another example for translational repression by Hfq entails regulation of the *E. coli sdhC* mRNA by the sRNA Spot 42. Desnoyers and Massé [48] reported that Spot 42 binds upstream of the RBS and recruits Hfq, which then directly represses translation. Moreover, *E. coli* Hfq was recently shown to act as a repressor of *cirA* mRNA translation in the absence of a sRNA [49]. Interestingly, the translational block exerted by Hfq was shown to be abrogated by RyhB RNA pairing to *cirA* mRNA [49].

Similarly, the model shown in Figure 7 entails direct repression of target mRNAs by PAO1 Hfq. However, in contrast to Spot 42 [48] and RyhB [49] RNA, which recruit Hfq to and abrogate Hfq-mediated translational repression of the target mRNA, respectively, CrcZ RNA acts as a decoy for Hfq. The CrcZ RNA contains six (A-R-N)_n_ repeats (n≥4) (Figure S6A) that can potentially be exploited for binding to the distal face of Hfq. Thus, in the absence of CCR Hfq is most likely sequestered by CrcZ, and therefore not available to act as a translational repressor on target mRNAs. In this way the Hfq/CrcZ regulatory system is reminiscent to the *Pseudomonas* RsmA/RsmY/RsmZ system and the CsrA/CsrB system of several Gram-negative Bacteria [30], [50], [51]. The ability of Hfq to bind to RsmY [31] in fact poses the question whether the regulatory systems are interlinked. This could provide an explanation why different carbon sources can affect virulence traits such as biofilm formation and antibiotic resistance [39], [52].

Moreover, given its constellation with Hfq, it seems possible that CrcZ on the one hand can interfere with any mRNA-dependent role of Hfq. On the other hand, the role of Hfq in sRNA-mediated riboregulation remains ill defined in PAO1. Although Hfq appears to be required for the PAO1 sRNAs PrrF1/PrrF2 to regulate target mRNAs [53], it is unclear whether Hfq exerts this function by stabilizing the sRNAs and/or by stimulating sRNA-mRNA pairing. As CrcZ binds to the distal site of Hfq it is conceivable that Hfq might even still be able to bind sRNAs such as PrrF at the proximal site, and thus to protect them from degradation. In any case it will be interesting to study whether sequestration of Hfq by CrcZ indirectly affects other Hfq-mediated processes in *P. aeruginosa*.

### The elusive function of Crc in CCR of *Pseudomonas*

We have recently shown that the Crc protein is devoid of RNA binding activity [10], and that the previously observed RNA binding activity of Crc could be attributed to Hfq [11]. However, Crc contributes to Hfq-mediated repression during CCR (Figure 1). In addition, Crc was shown to impact on biofilm formation [54], [55], virulence and antibiotic susceptibility [52], traits which are also affected by Hfq [31], [33]. This raises the question how Crc impacts on Hfq function. Crc appears not to interfere with *hfq* expression. The translation of a *hfq::lacZ* reporter gene, whose transcription is driven by the authentic *hfq* promoter, was indistinguishable in PAO1 and in the isogenic PAO1Δ*crc* strain (Figure S10A). In addition, the levels of Hfq, as determined by quantitative western-blot analysis, were not significantly altered in PAO1Δ*crc* when compared with the wild-type strain (Figure S10B). Conversely, Hfq seems not to affect the cellular concentration of Crc (Figure S10B). Thus, Crc seems not to impact on the Hfq levels, and *vice versa* Hfq seems not to affect that of Crc.

RelA was recently shown to enhance multimerization of *E. coli* Hfq, and thereby to stimulate binding to sRNAs [56]. We therefore considered the possibility that Crc might interact with Hfq to increase the specificity of Hfq for A-rich sequences. However, as revealed by EMSA assays, the presence of Crc did not increase the affinity of PAO1 Hfq for *amiE*_−134–+20_ RNA (E. Sonnleitner, unpublished), making it less likely that Crc acts similar to RelA. Nevertheless, we are currently exploring the possibility whether Crc is associated with Hfq *in vivo*.

## Materials and Methods

### Bacterial strains, plasmids and growth conditions

The strains and plasmids used in this study are listed in Table S2. Unless indicated otherwise, the cultures were grown at 37°C in BSM minimal medium supplemented with 40 mM succinate. The strains PAO1*hfq*-Δ*crc* and PAO1*hfq*-Δ*crcZ* were constructed by homologous recombination. Briefly, plasmid pME9672 and plasmid pME9673, respectively, were mobilized into strain PAO1*hfq*- with the aid of *E. coli* strain HB101(pRK2013), and then chromosomally integrated through selection for tetracycline resistance. Excision of the vector by a second crossover event was achieved by enrichment for tetracycline-sensitive cells [57]. If required *E. coli* and PAO1 were grown in the presence of 100 µg ml^−1^ ampicillin, 25 µg ml^−1^ tetracycline or 25 µg ml^−1^ kanamycin and 50 µg ml^−1^ gentamicin, 100 µg ml^−1^ tetracycline or 250 µg ml^−1^ carbenicillin, respectively. Details on the construction of plasmids used in this study are provided in Text S1.

### β-Galactosidase assays

The β-galactosidase activities were determined as described by Miller [58]. The cells were permeabilized with 5% toluene. The β-galactosidase units in the different experiments were derived from three independent experiments. The error bars in the different Figures represent standard deviations.

### Western-blot analyses

The protein levels of Hfq and Crc fused to C-terminal Flag-tags (DYKDDDDK) were determined in the respective strains and under the growth conditions as specified in the legends to the Figures. 1 ml aliquots of the respective cultures were withdrawn; the cells were harvested by centrifugation, resuspended and boiled in protein sample buffer. Equal amounts of total protein were separated on 12% SDS-polyacrylamide gels and then electro-blotted to a nitrocellulose membrane. The blots were blocked with 5% dry milk in TBS buffer, and then probed with rabbit anti-DYKDDDDK polyclonal antibody (Roth). The antibody-antigen complexes were visualized with alkaline-phosphatase conjugated secondary antibodies (Sigma) using the chromogenic substrates nitro blue tetrazolium chloride (NBT) and 5-Bromo-4-chloro-3-indolyl phosphate (BCIP).

### Protein purification

Hfq protein, Hfq_Y25D_ and Hfq_K56A_ protein were produced in the *hfq* deficient *E. coli* strain AM111F′ harboring plasmid pHfq_Pae_, pHfq_PaeY25D_ or pHfq_PaeK56A_. Protein purifications were performed as described in detail by Beich-Frandsen et al. [59].

### *In vitro* transcription

For *in vitro* transcription of *amiE* (1172 nt), CrcZ (426 nt) and RsmZ (141 nt) RNAs the AmpliScribe T7-Flash Transcription Kit (Epicentre Biotechnologies) was used according to the manufacturer's instructions. First, PCR fragments were generated with the primer pairs (see Table S3) A5/A75 (*amiE*), E6/C6 (*crcZ*) and W26/X26 (*rsmZ*), whereby the forward primers contained T7 promoter sequences. Primer A75 encoded in addition a Flag-tag sequence. For the filter binding and gel mobility shift assays truncated versions of *amiE*_−134–+20_ (first 154 nt) and *crcZ*_151_ (first 151 nt) were used. The corresponding PCR fragments were amplified with the primers A5/C1 (*amiE*_−134–+20_) and E6/E2 (*crcZ*_151_). For the toeprint assay the *amiE*_−134–+76_ fragment was *in vitro* transcribed using oligonucleotides A5 and Q99 (Table S3).

### Enzymatic probing of RNA

Five pmol of *in vitro* transcribed full length *amiE* RNA was incubated in RT-buffer (50 mM Tris pH 8.3, 60 mM NaCl, 6 mM Mg-acetate, 10 mM DTT) at 37°C for 30 min in a 10 µl reaction with 0, 5 and 20 pmol of purified Hfq protein. Then 2 U RNase T1 (1 µl), 10 pg RNase A (1 µl) or 1 µl of RNase free H_2_O was added and incubated for additional 10 min followed by phenol/chloroform extraction and precipitation. 200 fmol RNase treated or untreated RNAs were further used for the primer extension reaction with AMV reverse transcriptase (Promega), which was primed with the 5′-[^32^P]-labeled C78 oligonucleotide to test for protection by Hfq of the proximal part of *amiE* mRNA. For sequencing *amiE* RNA and ddNTPs (Fermentas) were used in primer extension reaction(s).

### Toeprint analysis

The *amiE*_−134–+76_ RNA used for toeprinting was obtained as described above. The [^32^P]-5′-end labeled oligonucleotide Q99 was annealed to *amiE* mRNA (+57 to +76 with regard to the A (+1) of the start codon) and used to prime cDNA synthesis by MMuLV reverse transcriptase (Thermo Scientific). The toeprinting assay was carried out with purified *E. coli* 30S ribosomal subunits and *E. coli* initiator-tRNA (tRNA^fMet^) as described by Hartz et al. [37]. The mRNA (0.05 pmol) was pre-incubated at 37°C for 10 min with or without 4 pmol 30S subunits and 16 pmol tRNA^fMet^ before reverse transcriptase was added. To test whether Hfq interferes with translation initiation, 0.05 pmol *amiE*_−134–+76_ mRNA was pre-incubated at 37°C for 10 min with 4 pmol 30S subunits, 16 pmol tRNA^fMet^ and Hfq-hexamer (1 or 2 pmol, respectively) before the reverse transcriptase reaction was performed. To test whether CrcZ can abrogate the Hfq mediated repression of *amiE* translation initiation, 0.05 pmol *amiE*_−134–+76_ mRNA was pre-incubated at 37°C for 10 min with 4 pmol 30S subunits, 16 pmol tRNA^fMet^ and equimolar amounts of Hfq-hexamer and CrcZ (1 or 2 pmol, respectively) before reverse transcriptase was added.

### Electro mobility shift assays

The *amiE*_−134–+20_ and *crcZ*_151_ RNAs (see above) were dephosphorylated with FastAP thermo sensitive alkaline phosphatase (Thermo Scientific) and subsequently 5′-end labeled using [γ-^32^P]-ATP (Hartmann Analytic) and polynucleotide kinase (Thermo Scientific). The labeled RNAs were gel-purified and dissolved in diethylpyrocarbonate-treated water. Labeled RNA (10 nM) was incubated with increasing amounts of purified Hfq, the Hfq_Y25D_ or Hfq_K56A_ mutant proteins in 10 mM Tris-HCl (pH 8.0), 10 mM MgCl_2_, 60 mM NaCl, 10 mM NaH_2_PO_4_, 10 mM DTT, and 25 ng tRNA in a total volume of 10 µl. Unlabeled RNA was used as competitor as stated in the legend to Figure 5A. The reaction mixtures were incubated at 37°C for 30 min to allow protein–RNA complex formation. The samples were mixed with 4 µl loading dye (25% glycerol, 0.2 mg/l xylencyanol and bromphenol blue) immediately before loading and separated on 4% polyacrylamide gels using Tris-borate buffer. The radioactively labeled bands were visualized with a PhosphorImager (Molecular Dynamics) and quantified with ImageQuant software 5.2.

### *In vitro* translation

*In vitro* translation was performed with the PURExpress *in vitro* protein synthesis kit (New England BioLabs). 5 pmol *in vitro* transcribed *amiE*_Flag_ mRNA was used in a 12.5 µl reaction. Increasing amounts of purified Hfq protein (as specified in legends of Figures 5B and S4) were added. For competition CrcZ (5 and 10 pmol) or RsmZ (10 pmol) RNA were added. After 1 h of incubation at 37°C, 5 µl of the reaction was mixed with 5 µl protein loading buffer and a western-blot was performed using anti-Flag antibodies as described above.

### Esterase assay

Esterase activity was assayed as described previously [60]. Briefly, the cells were harvested by centrifugation and washed in 100 mM potassium phosphate buffer pH 7.2. The substrate (25 µl *p*-nitrophenyl-caproate dissolved in 5 ml ethanol) was added to 100 ml potassium phosphate buffer (100 nM; pH 7.2) containing MgSO_4_ to a final concentration of 10 mM. 1 ml of the test solution and 50 µl of cells were used to determine esterase activity by monitoring the change in absorbance at 410 nM min^−1^, which was normalized to the optical density (OD_600_) of the culture.

### Northern-blot analyses

Total RNA of the respective strains as specified in the legends to the Figures was purified using hot phenol. The steady state levels of CrcZ and 5S rRNA (loading control) was determined by Northern-blotting using 4 µg of total RNA. The RNA samples were denatured for 5 min at 65°C in loading buffer containing 50% formamide, separated on a 8% polyacrylamide/8 M urea gel, and then transferred to a nylon membrane by electroblotting. The RNAs were cross-linked to the membrane by exposure to UV light. The membranes were hybridized with gene-specific ^32^P-end-labelled oligonucleotides (CrcZ: K3; 5S rRNA: I26; Table S3). The hybridization signals were visualized using a PhosphorImager (Molecular Dynamics).

## Supporting Information

Figure S1 CCR represses *amiE* at the post-transcriptional level. (**A**) The strains were grown to an OD_600_ of 2.0 in BSM medium supplemented with 40 mM succinate and 40 mM acetamide (blue bar; CCR) or with 40 mM mannitol and 40 mM acetamide (grey bar; no CCR). Acetamide was added to induce transcription of the chimeric *amiE* genes. Then, the cells were harvested and the β-galactosidase activities were determined. The bars depict the β-galactosidase values conferred by the transcriptional *amiE-lacZ* fusion encoded by plasmid pTC*amiE* (**left panel**) and by the translational *amiE::lacZ* fusion encoded by plasmid pME9655 (**right panel**) in strains PAO1(pTC*amiE*) and PAO1(pME9655), respectively. (**B**) The strains were grown to an OD_600_ of 2.0 in BSM medium supplemented with 40 mM succinate and 40 mM acetamide. Then, the cells were harvested and the β-galactosidase activities were determined. The bars depict the β-galactosidase values conferred by the transcriptional *amiE-lacZ* fusion encoded by plasmid pTC*amiE* in strains PAO1 (blue bar), PAO1Δ*crc* (green bar), PAO1*hfq*- (red bar) and PAO1*hfq*-Δ*crc* (purple bar), respectively. (**C**) Hfq does not affect *lacZ* expression. The strains were grown to an OD_600_ of 2.0 in BSM medium supplemented with 40 mM succinate and 1 mM IPTG. Then, the cells were harvested and the β-galactosidase activities were determined. The bars depict the β-galactosidase values conferred by the *lacZ* gene encoded by plasmid pME3856 in strains PAO1 (blue bar), PAO1Δ*crc* (green bar), PAO1*hfq*- (red bar) and PAO1*hfq*-Δ*crc* (purple bar), respectively. The error bars represent standard deviations from three independent experiments.
(TIF)

Figure S2 Hfq binds to *amiE* mRNA and CrcZ RNA *in vivo* during mimicked CCR. Strains PAO1*hfq*-(pMMB*hfq*_Flag_) and PAO1*hfq*-(pMMB67HE) (control) were grown under conditions of mimicked CCR in BSM medium supplemented with 40 mM succinate, 40 mM acetamide (transcriptional induction of *amiE*) and 1 mM IPTG (transcriptional induction of *hfq*_Flag_). Then, lysates were prepared and RNAs associated with Hfq were co-immunoprecipitated with Hfq specific antibodies. RNA was extracted from the co-immunoprecipitate and from the remaining supernatant. Equal concentrations were used for RT-PCR with specific primers for *amiE*, CrcZ and RsmZ (control RNA) as described in Text S1. Lanes 1 and 2, RT-PCR with *amiE*, CrcZ and RsmZ specific oligonucleotides performed with RNA obtained from the supernatant after co-immunoprecipitation (S; not in complex with Hfq_Flag_) and with RNA obtained after CoIP with Hfq specific antibodies (CoIP; in complex with Hfq) in lysates of strain PAO1*hfq-*(pMMB*hfq*_Flag_). Lanes 3 and 4, RT-PCR with *amiE*, CrcZ and RsmZ specific oligonucleotides performed with RNA obtained from the supernatant after co-immunoprecipitation (S) and after mock co-immunoprecipitation (CoIP) in the absence of Hfq in strain PAO1*hfq*-(pMMB67HE). Lane 5, chromosomal DNA of PAO1 served as a positive control. Lane 6, “RT-PCR” reaction without reverse transcriptase (negative control; -RT) with RNA obtained after CoIP with Hfq-specific antibodies, i.e. as used in the reactions shown in lane 2.
(TIF)

Figure S3 Filter binding assay of *amiE*_−134–+20_ with increasing amounts of Hfq-hexamer. The percentage of bound RNA is blotted against the concentration of Hfq-hexamer. The experiment was performed in duplicate.
(TIF)

Figure S4 Repression of *amiE*_Flag_ mRNA translation in the presence of Hfq. Lane 1, *in vitro* translation of *amiE*_Flag_ mRNA. Lanes 2–7, inhibition of *amiE*_Flag_ mRNA translation in the presence of increasing amounts of Hfq. The molar ratios of Hfq hexamer (Hfq_6_) to *amiE*_Flag_ mRNA are denoted on top.
(TIF)

Figure S5 (**A**) EMSA with 10 nM radioactively labeled *amiE*_−134–+20_ RNA in the presence of increasing amounts of Hfq, Hfq_Y25D_ and Hfq_K46A_. Lane 1, labeled *amiE*_−134–+20_ RNA without addition of protein. Lanes 2–4, lanes 5–7 and lanes 8–10, Hfq, Hfq_Y25D_ and Hfq_K56A_ were added in 5, 10 and 20-fold molar excess over *amiE*_−134–+20_ RNA, respectively. (**B**) Repression of *amiE* translation by PAO1 Hfq in *E. coli*. The strain JW4130(pME9658) concomitantly harboring the control plasmid pME4510 (red bar), plasmid pME4510*hfq*_Flag_ (blue bar), plasmid pME4510*hfq*_Y25DFlag_ (orange bar) and pME4510*hfq*_K56AFlag_ (purple bar), respectively, was grown in LB broth to an OD_600_ of 2.0. Then, the β-galactosidase activity conferred by the translational *amiE*_Δterm_ *::lacZ* fusion encoded by plasmid pME9658 was determined. Inset: the levels of the PAO1 Hfq_Flag_, PAO1 Hfq_Y25DFlag_ and PAO1 Hfq_K56AFlag_ proteins were determined by quantitative western-blot analysis using anti-Flag antibodies. The error bars represent standard deviations from three independent experiments.
(TIF)

Figure S6 Hfq binds to CrcZ with its distal face. (**A**) Schematic representation of CrcZ RNA. Potential Hfq binding sites are depicted in red. (**B**) EMSA with 10 nM radioactively labeled CrcZ_151_ RNA in the presence of increasing amounts of Hfq, Hfq_Y25D_ and Hfq_K56A_. Lane 1, labeled CrcZ_151_ RNA without addition of protein. Lanes 2 and 3, lanes 4 and 5 and lanes 6 and 7, Hfq, Hfq_Y25D_ and Hfq_K56A_ were added in 5 and 20-fold molar excess over CrcZ_151_ RNA, respectively.
(TIF)

Figure S7 Hfq is required for CrcZ function. The strains were grown to an OD_600_ of 0.5 in BSM medium supplemented with 40 mM succinate and 40 mM acetamide. Then IPTG was added (1 mM final concentration). At an OD_600_ of 2.0 the cells were harvested and the β-galactosidase activities were determined. The bars depict the β-galactosidase values conferred by the translational *amiE::lacZ* fusion encoded by plasmid pME9655 in strain PAO1 harboring either the control plasmid pMMB67HE (blue bar) or the *crcZ* encoding plasmid pMMB*crcZ* (pink bar) (**A**) and in strain PAO1*hfq*- harboring either the control plasmid pMMB67HE (red bar) or the *crcZ* encoding plasmid pMMB*crcZ* (pink bar) (**B**), respectively. The error bars represent standard deviations from three independent experiments. The CrcZ levels (top panel) were determined by Northern-blot analysis. 5S rRNA served as a loading control.
(TIF)

Figure S8 CCR results in destabilization of *amiE* mRNA. PAO1 was grown in BSM medium supplemented with 40 mM acetamide (No CCR) or supplemented with 40 mM acetamide and 40 mM succinate (CCR). PAO1*hfq*- was grown in BSM medium supplemented with 40 mM acetamide and 40 mM succinate (CCR). At an OD_600_ of 1.0, rifampicin was added to a final concentration of 100 µg/ml and samples were withdrawn for total RNA extraction at the times indicated. (**A**) The remaining levels of *amiE* and 16S rRNA (control) were determined by RT-PCR with oligonucleotides specific for either RNA as described in Text S1. The result from one representative experiment is shown. (**B**) The amounts of *amiE* mRNA during CCR in PAO1 (blue squares) and in PAO1*hfq-* (red triangles), respectively, as well as in PAO1 in the absence of CCR (green diamonds) were normalized to that of 16S rRNA at different times after addition of rifampicin. The results are derived from three independent experiments. Error bars represent standard deviations. The half-life of *amiE* mRNA was determined with 6.8+/−0.4 min in PAO1 and with 10.1+/−0.5 min in PAO1*hfq-* during CCR. In the absence of CCR the half-life of *amiE* mRNA was determined with 11.4+/−0.8 min.
(TIF)

Figure S9 Determination of the cellular Hfq concentration. PAO1 was grown in BSM medium supplemented with 40 mM succinate to an OD_600_ of 2.0. ( = 3.2±0.3 10^9^ CFU/ml). The Hfq concentration was determined in triplicate samples of PAO1 cell lysates corresponding to 50 µl of culture (lanes 2–4) using quantitative western-blotting with Hfq specific antibodies. Lane 1, marker protein. Lanes 5–7, 0.1, 0.4 and 0.6 pmol of purified Hfq_6_ protein were loaded, respectively. The Hfq_6_ concentration per cell was determined as described in Text S1.
(TIF)

Figure S10 Crc does not affect *hfq* expression. (**A**) Determination of the β-galactosidase activity conferred by a translational *hfq::lacZ* fusion encoded by plasmid pTL*hfq* during growth in strain PAO1 (blue squares) and in strain PAO1Δ*crc* (green circles). The strains were grown in BSM medium supplemented with 40 mM succinate. The error bars represent standard deviations from three independent experiments. (**B**) Levels of Hfq and Crc in PAO1 (wt), PAO1Δ*crc* (Δ*crc*) and PAO1*hfq*- (Δ*hfq*) grown to an OD_600_ of 2.0 in BSM medium supplemented with 40 mM succinate. Immunodetection of Hfq, Crc and of ribosomal protein S2 (loading control) was performed as described in Text S1.
(TIF)

Table S1 Catabolic and transport genes that are up-regulated in the absence of Hfq.
(DOCX)

Table S2 Strains and plasmids used in this study.
(DOCX)

Table S3 Oligonucleotides used in this study.
(DOCX)

Text S1 Supporting materials and methods and references.
(DOCX)
