# Supplementary figures and images for: Regulation of Hfq by the RNA CrcZ in Pseudomonas aeruginosa Carbon Catabolite Repression
Source: PLoS Genet. 2014 Jun 19;10(6):e1004440. doi: 10.1371/journal.pgen.1004440 (PMC4063720; doi:10.1371/journal.pgen.1004440)

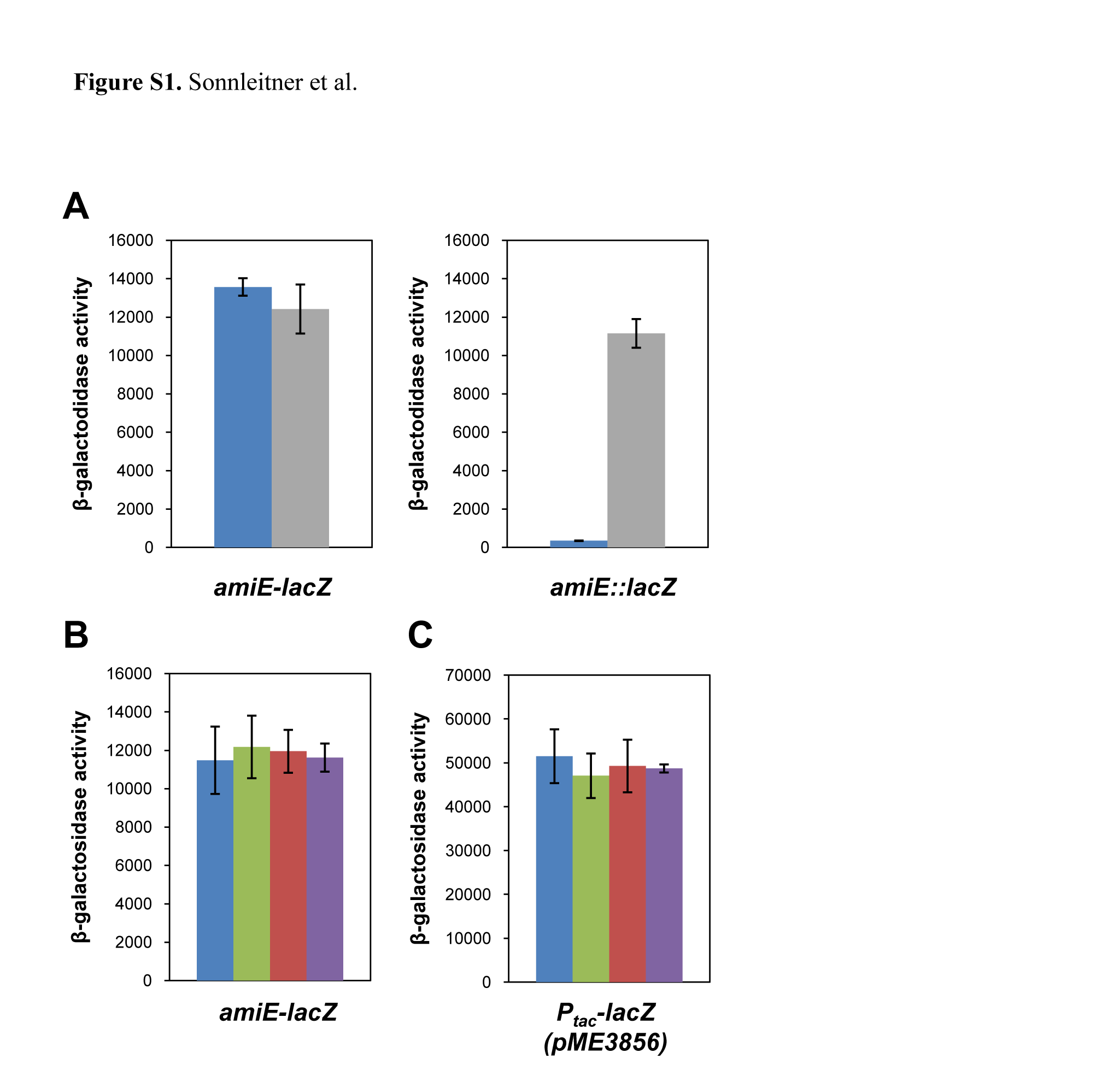

Supplement: Figure S1 — CCR represses amiE at the post-transcriptional level. (A) The strains were grown to an OD600 of 2.0 in BSM medium supplemented with 40 mM succinate and 40 mM acetamide (blue bar; CCR) or with 40 mM mannitol and 40 mM acetamide (grey bar; no CCR). Acetamide was added to induce transcription of the chimeric amiE genes. Then, the cells were harvested and the β-galactosidase activities were determined. The bars depict the β-galactosidase values conferred by the transcriptional amiE-lacZ fusion encoded by plasmid pTCamiE (left panel) and by the translational amiE::lacZ fusion encoded by plasmid pME9655 (right panel) in strains PAO1(pTCamiE) and PAO1(pME9655), respectively. (B) The strains were grown to an OD600 of 2.0 in BSM medium supplemented with 40 mM succinate and 40 mM acetamide. Then, the cells were harvested and the β-galactosidase activities were determined. The bars depict the β-galactosidase values conferred by the transcriptional amiE-lacZ fusion encoded by plasmid pTCamiE in strains PAO1 (blue bar), PAO1Δcrc (green bar), PAO1hfq- (red bar) and PAO1hfq-Δcrc (purple bar), respectively. (C) Hfq does not affect lacZ expression. The strains were grown to an OD600 of 2.0 in BSM medium supplemented with 40 mM succinate and 1 mM IPTG. Then, the cells were harvested and the β-galactosidase activities were determined. The bars depict the β-galactosidase values conferred by the lacZ gene encoded by plasmid pME3856 in strains PAO1 (blue bar), PAO1Δcrc (green bar), PAO1hfq- (red bar) and PAO1hfq-Δcrc (purple bar), respectively. The error bars represent standard deviations from three independent experiments. (TIF) [file pgen.1004440.s001.tif]

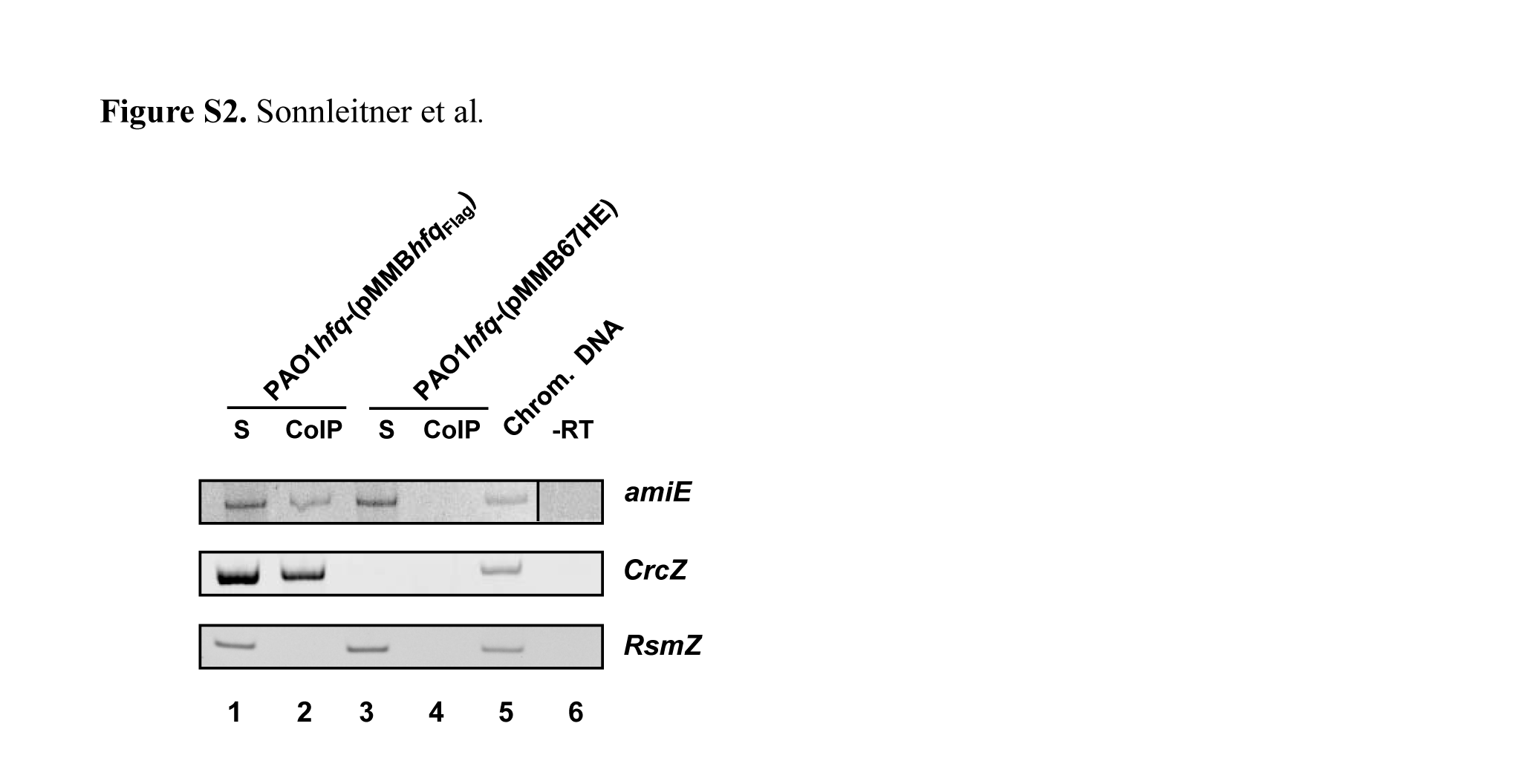

Supplement: Figure S2 — Hfq binds to amiE mRNA and CrcZ RNA in vivo during mimicked CCR. Strains PAO1hfq-(pMMBhfq Flag) and PAO1hfq-(pMMB67HE) (control) were grown under conditions of mimicked CCR in BSM medium supplemented with 40 mM succinate, 40 mM acetamide (transcriptional induction of amiE) and 1 mM IPTG (transcriptional induction of hfq Flag). Then, lysates were prepared and RNAs associated with Hfq were co-immunoprecipitated with Hfq specific antibodies. RNA was extracted from the co-immunoprecipitate and from the remaining supernatant. Equal concentrations were used for RT-PCR with specific primers for amiE, CrcZ and RsmZ (control RNA) as described in Text S1. Lanes 1 and 2, RT-PCR with amiE, CrcZ and RsmZ specific oligonucleotides performed with RNA obtained from the supernatant after co-immunoprecipitation (S; not in complex with HfqFlag) and with RNA obtained after CoIP with Hfq specific antibodies (CoIP; in complex with Hfq) in lysates of strain PAO1hfq-(pMMBhfq Flag). Lanes 3 and 4, RT-PCR with amiE, CrcZ and RsmZ specific oligonucleotides performed with RNA obtained from the supernatant after co-immunoprecipitation (S) and after mock co-immunoprecipitation (CoIP) in the absence of Hfq in strain PAO1hfq-(pMMB67HE). Lane 5, chromosomal DNA of PAO1 served as a positive control. Lane 6, “RT-PCR” reaction without reverse transcriptase (negative control; -RT) with RNA obtained after CoIP with Hfq-specific antibodies, i.e. as used in the reactions shown in lane 2. (TIF) [file pgen.1004440.s002.tif]

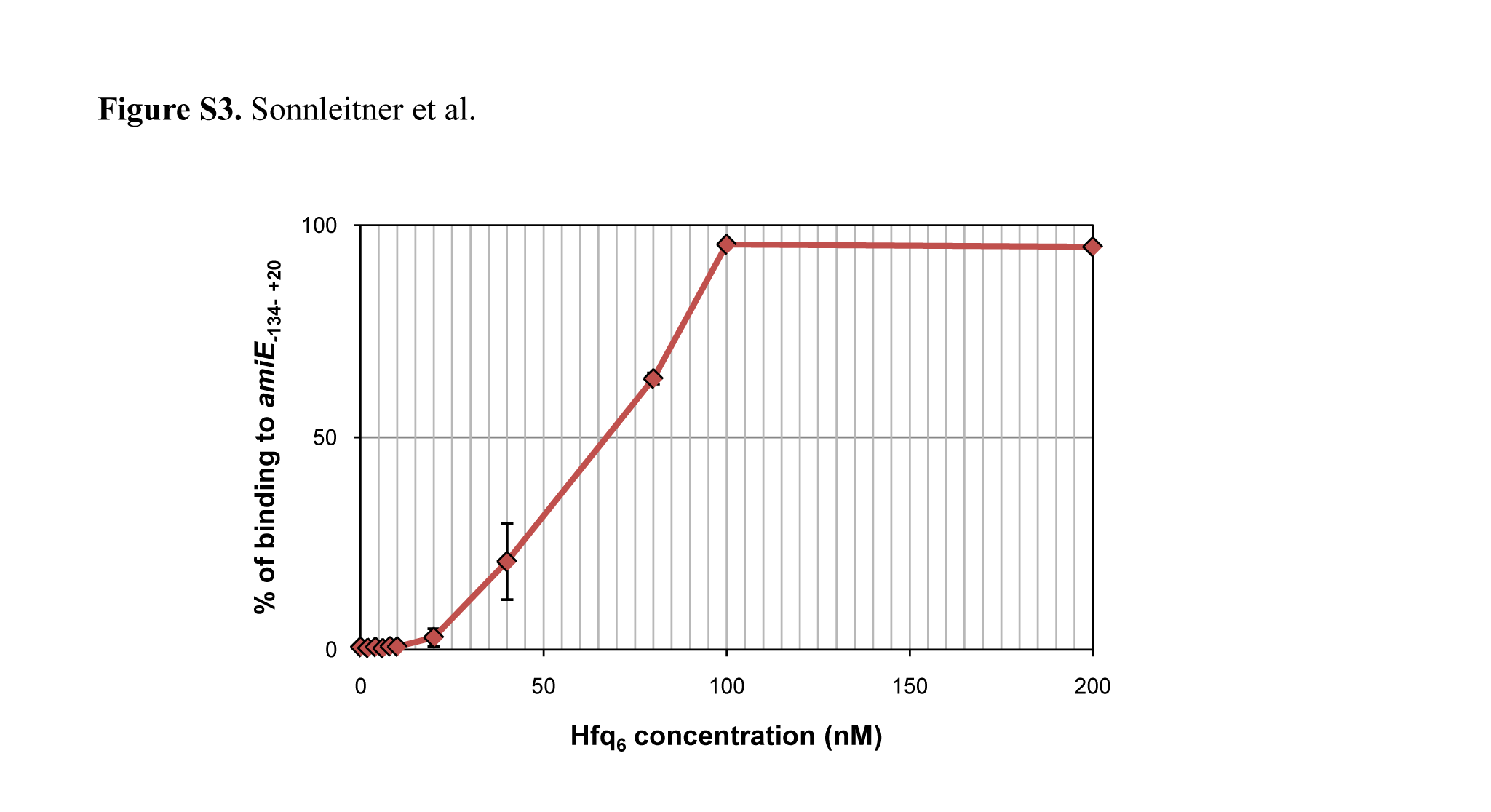

Supplement: Figure S3 — Filter binding assay of amiE −134–+20 with increasing amounts of Hfq-hexamer. The percentage of bound RNA is blotted against the concentration of Hfq-hexamer. The experiment was performed in duplicate. (TIF) [file pgen.1004440.s003.tif]

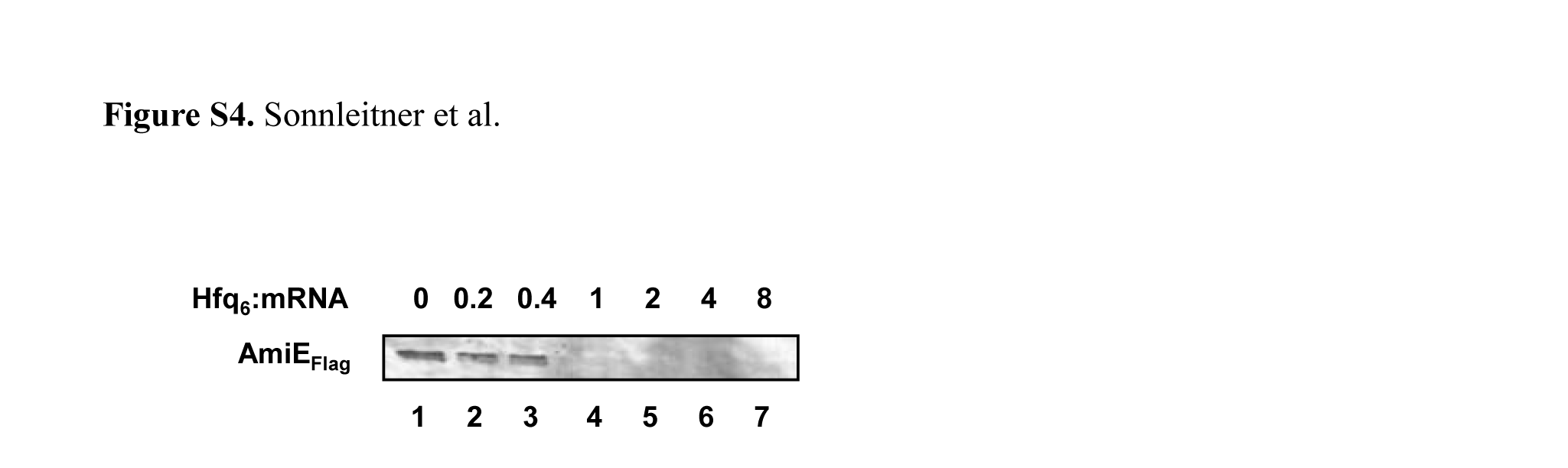

Supplement: Figure S4 — Repression of amiE Flag mRNA translation in the presence of Hfq. Lane 1, in vitro translation of amiE Flag mRNA. Lanes 2–7, inhibition of amiE Flag mRNA translation in the presence of increasing amounts of Hfq. The molar ratios of Hfq hexamer (Hfq6) to amiE Flag mRNA are denoted on top. (TIF) [file pgen.1004440.s004.tif]

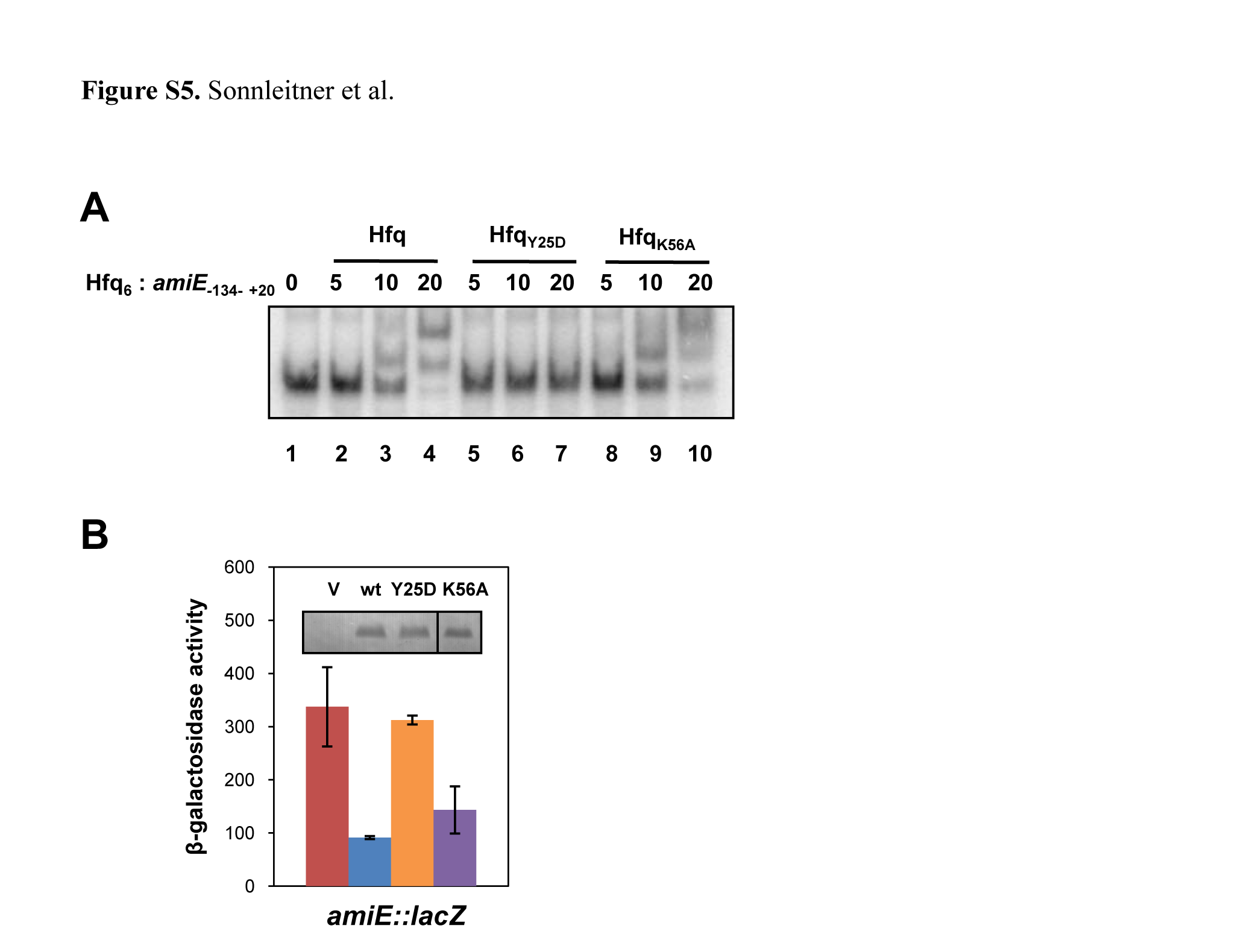

Supplement: Figure S5 — (A) EMSA with 10 nM radioactively labeled amiE −134–+20 RNA in the presence of increasing amounts of Hfq, HfqY25D and HfqK46A. Lane 1, labeled amiE −134–+20 RNA without addition of protein. Lanes 2–4, lanes 5–7 and lanes 8–10, Hfq, HfqY25D and HfqK56A were added in 5, 10 and 20-fold molar excess over amiE −134–+20 RNA, respectively. (B) Repression of amiE translation by PAO1 Hfq in E. coli. The strain JW4130(pME9658) concomitantly harboring the control plasmid pME4510 (red bar), plasmid pME4510hfq Flag (blue bar), plasmid pME4510hfq Y25DFlag (orange bar) and pME4510hfq K56AFlag (purple bar), respectively, was grown in LB broth to an OD600 of 2.0. Then, the β-galactosidase activity conferred by the translational amiE Δterm ::lacZ fusion encoded by plasmid pME9658 was determined. Inset: the levels of the PAO1 HfqFlag, PAO1 HfqY25DFlag and PAO1 HfqK56AFlag proteins were determined by quantitative western-blot analysis using anti-Flag antibodies. The error bars represent standard deviations from three independent experiments. (TIF) [file pgen.1004440.s005.tif]

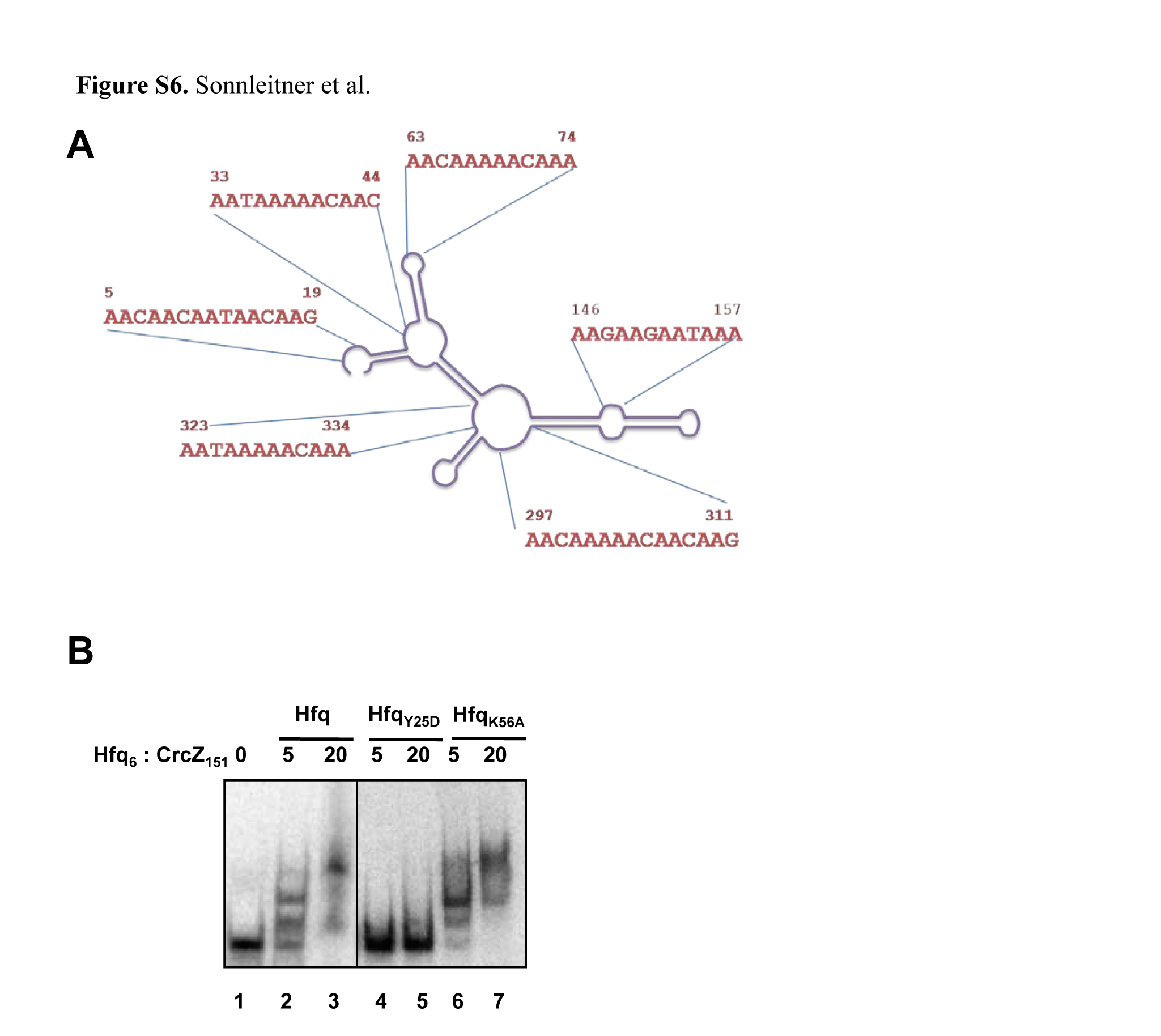

Supplement: Figure S6 — Hfq binds to CrcZ with its distal face. (A) Schematic representation of CrcZ RNA. Potential Hfq binding sites are depicted in red. (B) EMSA with 10 nM radioactively labeled CrcZ151 RNA in the presence of increasing amounts of Hfq, HfqY25D and HfqK56A. Lane 1, labeled CrcZ151 RNA without addition of protein. Lanes 2 and 3, lanes 4 and 5 and lanes 6 and 7, Hfq, HfqY25D and HfqK56A were added in 5 and 20-fold molar excess over CrcZ151 RNA, respectively. (TIF) [file pgen.1004440.s006.tif]

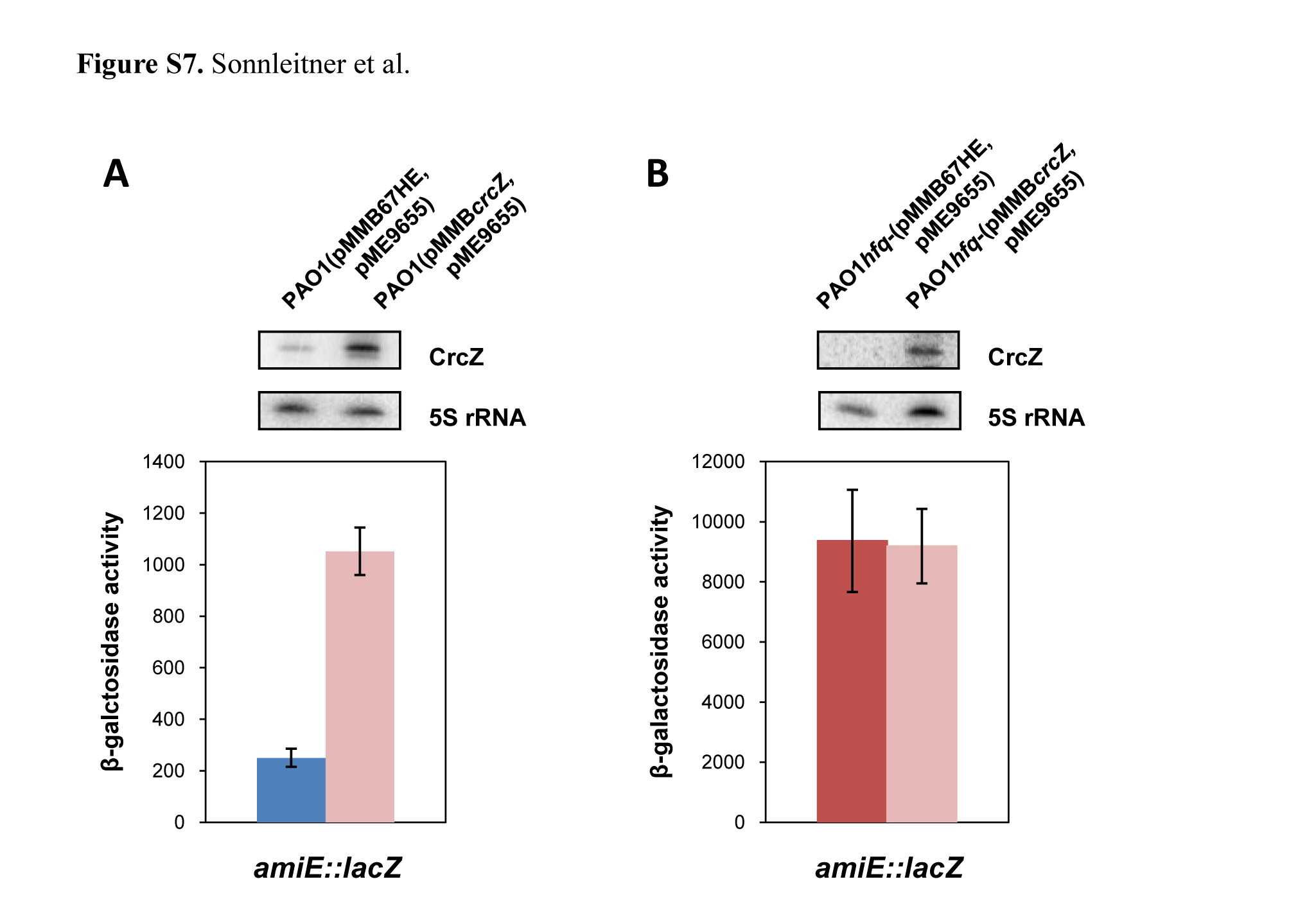

Supplement: Figure S7 — Hfq is required for CrcZ function. The strains were grown to an OD600 of 0.5 in BSM medium supplemented with 40 mM succinate and 40 mM acetamide. Then IPTG was added (1 mM final concentration). At an OD600 of 2.0 the cells were harvested and the β-galactosidase activities were determined. The bars depict the β-galactosidase values conferred by the translational amiE::lacZ fusion encoded by plasmid pME9655 in strain PAO1 harboring either the control plasmid pMMB67HE (blue bar) or the crcZ encoding plasmid pMMBcrcZ (pink bar) (A) and in strain PAO1hfq- harboring either the control plasmid pMMB67HE (red bar) or the crcZ encoding plasmid pMMBcrcZ (pink bar) (B), respectively. The error bars represent standard deviations from three independent experiments. The CrcZ levels (top panel) were determined by Northern-blot analysis. 5S rRNA served as a loading control. (TIF) [file pgen.1004440.s007.tif]

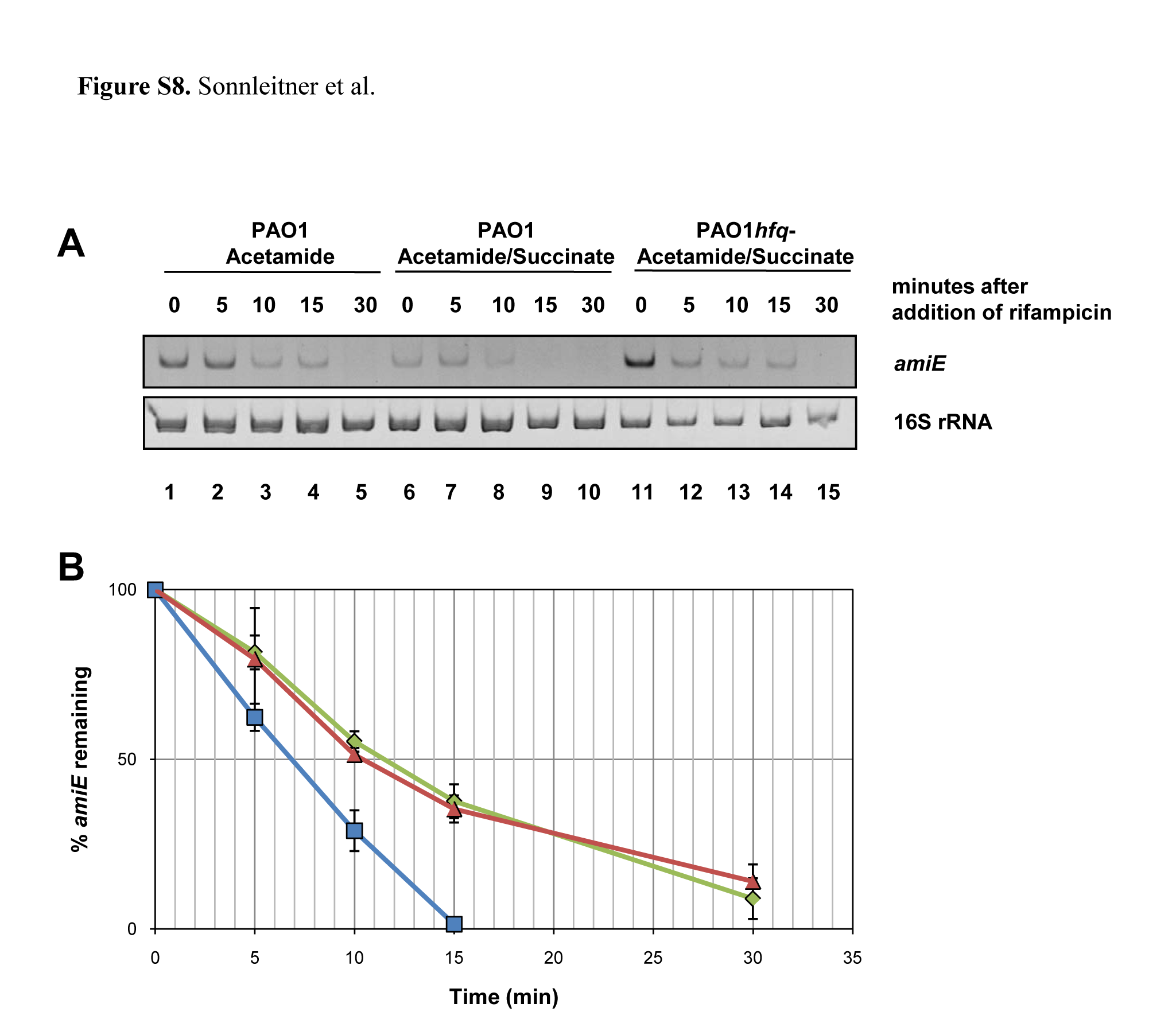

Supplement: Figure S8 — CCR results in destabilization of amiE mRNA. PAO1 was grown in BSM medium supplemented with 40 mM acetamide (No CCR) or supplemented with 40 mM acetamide and 40 mM succinate (CCR). PAO1hfq- was grown in BSM medium supplemented with 40 mM acetamide and 40 mM succinate (CCR). At an OD600 of 1.0, rifampicin was added to a final concentration of 100 µg/ml and samples were withdrawn for total RNA extraction at the times indicated. (A) The remaining levels of amiE and 16S rRNA (control) were determined by RT-PCR with oligonucleotides specific for either RNA as described in Text S1. The result from one representative experiment is shown. (B) The amounts of amiE mRNA during CCR in PAO1 (blue squares) and in PAO1hfq- (red triangles), respectively, as well as in PAO1 in the absence of CCR (green diamonds) were normalized to that of 16S rRNA at different times after addition of rifampicin. The results are derived from three independent experiments. Error bars represent standard deviations. The half-life of amiE mRNA was determined with 6.8+/−0.4 min in PAO1 and with 10.1+/−0.5 min in PAO1hfq- during CCR. In the absence of CCR the half-life of amiE mRNA was determined with 11.4+/−0.8 min. (TIF) [file pgen.1004440.s008.tif]

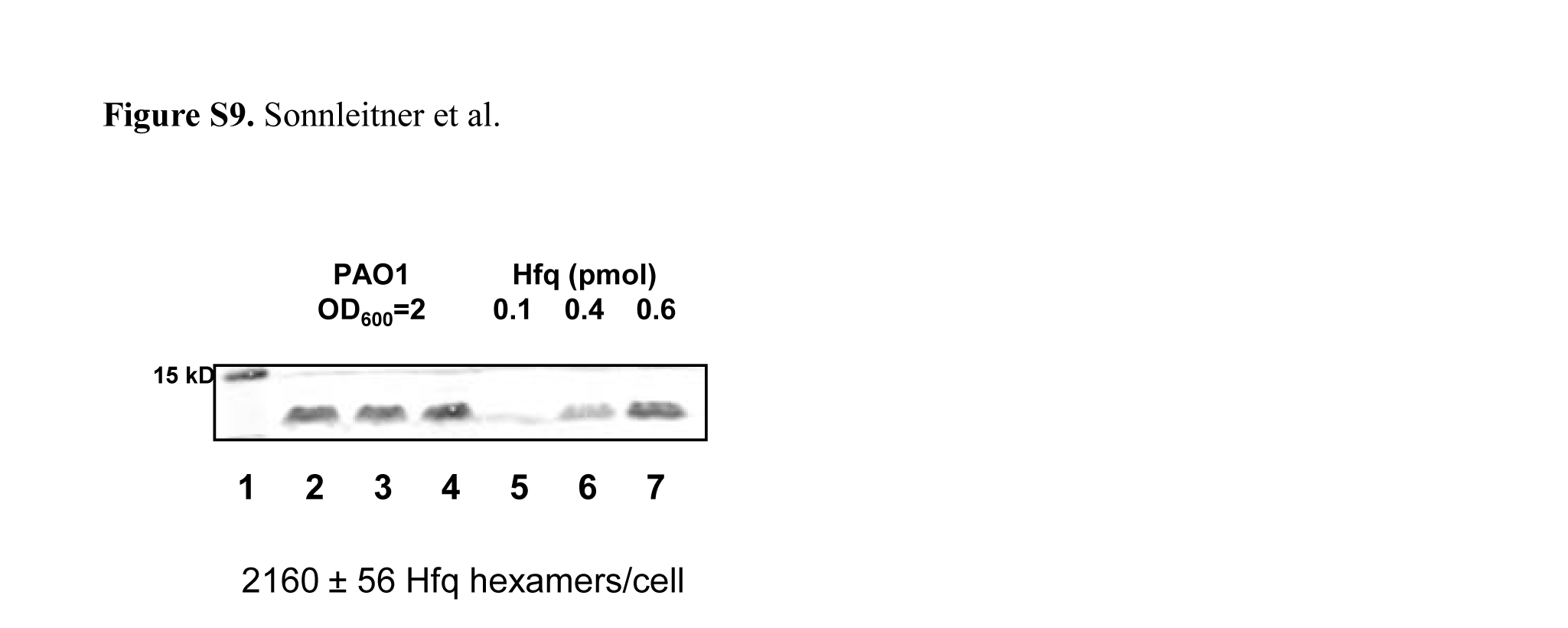

Supplement: Figure S9 — Determination of the cellular Hfq concentration. PAO1 was grown in BSM medium supplemented with 40 mM succinate to an OD600 of 2.0. ( = 3.2±0.3 109 CFU/ml). The Hfq concentration was determined in triplicate samples of PAO1 cell lysates corresponding to 50 µl of culture (lanes 2–4) using quantitative western-blotting with Hfq specific antibodies. Lane 1, marker protein. Lanes 5–7, 0.1, 0.4 and 0.6 pmol of purified Hfq6 protein were loaded, respectively. The Hfq6 concentration per cell was determined as described in Text S1. (TIF) [file pgen.1004440.s009.tif]

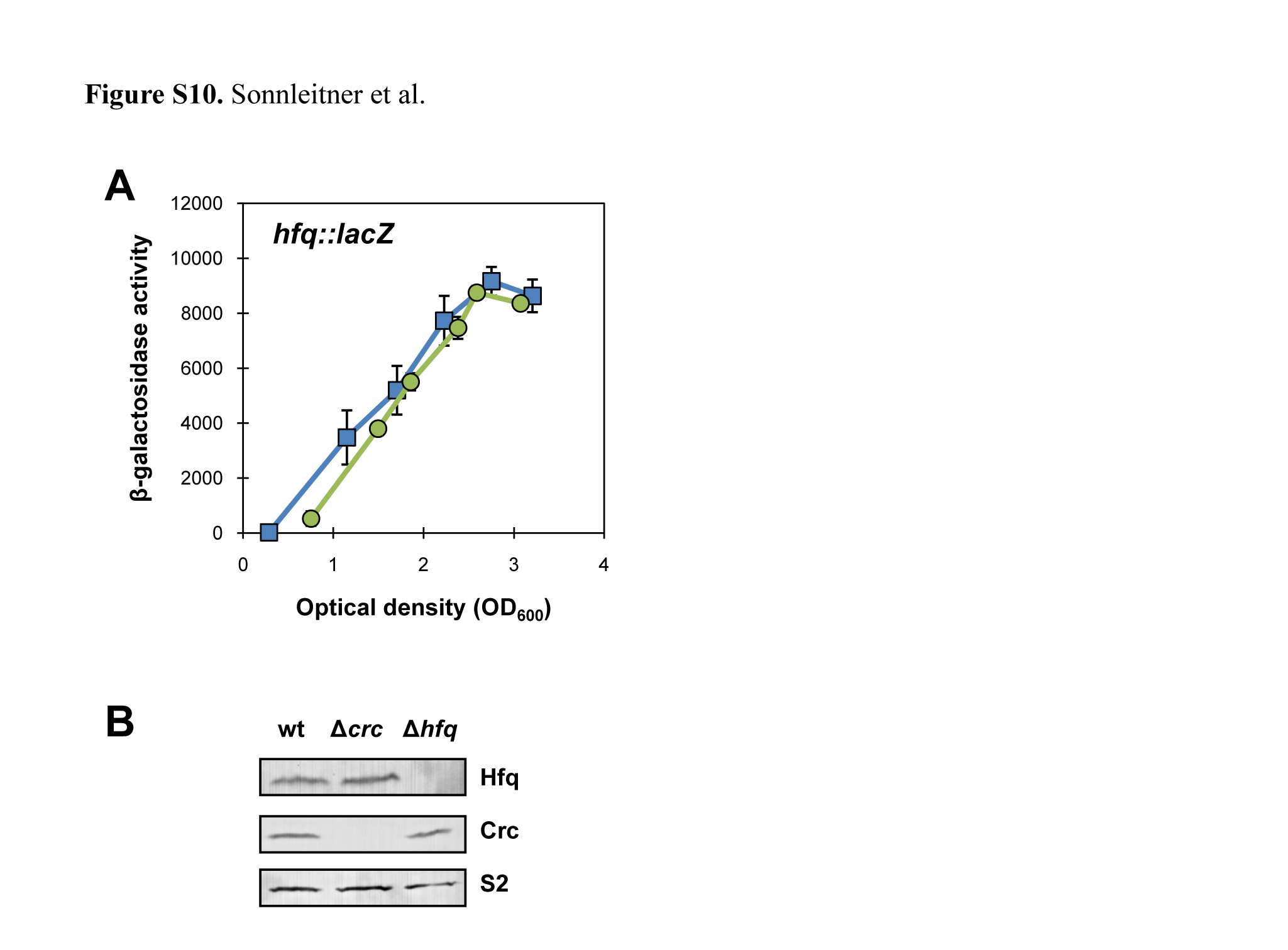

Supplement: Figure S10 — Crc does not affect hfq expression. (A) Determination of the β-galactosidase activity conferred by a translational hfq::lacZ fusion encoded by plasmid pTLhfq during growth in strain PAO1 (blue squares) and in strain PAO1Δcrc (green circles). The strains were grown in BSM medium supplemented with 40 mM succinate. The error bars represent standard deviations from three independent experiments. (B) Levels of Hfq and Crc in PAO1 (wt), PAO1Δcrc (Δcrc) and PAO1hfq- (Δhfq) grown to an OD600 of 2.0 in BSM medium supplemented with 40 mM succinate. Immunodetection of Hfq, Crc and of ribosomal protein S2 (loading control) was performed as described in Text S1. (TIF) [file pgen.1004440.s010.tif]
