# Supplementary material for: Regulation of Hfq by the RNA CrcZ in Pseudomonas aeruginosa Carbon Catabolite Repression
Source: PLoS Genet. 2014 Jun 19;10(6):e1004440. doi: 10.1371/journal.pgen.1004440 (PMC4063720; doi:10.1371/journal.pgen.1004440)
Supplement: Table S1 — Catabolic and transport genes that are up-regulated in the absence of Hfq. (DOCX) [file pgen.1004440.s011.docx]

**Table S1.** Catabolic and transport genes that are up-regulated in the absence of Hfq

| **ORF or operon**^a^ | **Gene** | **Function**^a^ | **Fold change *hfq*- *vs* wt**^b^ | **Predicted Hfq binding motifs**^c^ |
| --- | --- | --- | --- | --- |
| PA0119 |  | putative dicarboxylate transporter | 10 | **AATAAAAAT**GCGTTCATCGCCCGCCTCCGCG**AGG**CCCG**ATG**G**AAG**CGCCG**Aat**g |
| PA0129 | *bauD* | amino acid permease | 4.5 | **AAGAACAAT**T**AAA**GGGTCTCAACGatg |
| PA0153-PA0154 | *pcaHG* | protocatechuate degradation | 9.7 (*pcaH*),  4.0 (*pcaG*) | AT**AAC**GCG**AATAACAGGAGC**CCGACatg (*pcaH*) |
| PA0162 | *opdC* | histidine porin OpdC | 3.6 | **AAGAAG**G**AGCAAC**CGC**Aat**g**AGGAAT** |
| PA0226-PA0228 | *pcaF* | protocatechuate degradation | 11 | AC**AACAAG**ACG**AGA**CCCGACCGatg (PA0226) C**AGAAGG**GG**AGC**GACGAatg (*pcaF*) |
| PA0297-PA0299 | *spuABC* | glutamine biosynthesis | 7.6 (*spuA*), 5.6 (*spuB*), 6.4 (*spuC*) | AT**AAC**ATCTACA**AACAAC**GGGTGTCTCatg (*spuA*); **AATAGAAGA**GGTGTGACatg (*spuC*) |
| PA0300 | *spuD* | polyamine transport protein | 4.5 | C**AACAAAAAT**GGAGCTACCCCGCatg |
| PA0301-PA0304 | *spuEFGH* | polyamine transport proteins | 4.8 (*spuE*), 7.8 (*spuF*), 7.3 (*spuG*), 5.7 (*spuH*) | **AAAAGG**CCCGG**AACAGGAGT**CGGACatg (*spuE*) **AACAGC**CTG**AAAAAG**CGCatg (*spuG*) A**AAGAAC**TGG**AGG**GC**AAG**GTatg (*spuH*) |
| PA0439-PA0440 |  | putative oxidoreductases for glutamate biosynthesis | 129 (-39), 99 (-40) | **AAGAAA**CC**AGAAGA**G**AGG**CTAC**AAC**Cgtg (PA0440) |
| PA0441 | *dht* | dihydropyrimidinase | 45 | A**AAAAACAAT**TTC**AAGAAC**CGGC**AAC**GACCGGTC**AGC**CTGCG**AGGAAA**GACGGCatg |
| PA0443-PA0444 |  | putative transporter and N-carbamoyl-beta-alanine amidohydrolase | 95 (-43), 11 (-44) | **AATAAC**TAA**AAGAAT**TCTGG**AGA**GGCCCatgC**AACAGA**GC**AGA** (PA0443) |
| PA0783 | *putP* | sodium/proline symporter | 3.6 | AT**AAAAACAAG**C**AATAGG**GG**AGT**CCCTatg |
| PA0866 | *aroP2* | aromatic amino acid transport protein | 84 | AC**AACAATAAA**TTTCCACTTGCGTG**AGA**T**AAT**TCAatg |
| PA0870-PA0872 | *phhABC* | phenylalanine metabolism | 2.5 (*phhB*), 2.3 (*phhC*) | **AACAACAAA**GACCCCGCGGCCACAAGCCGCTCATGGAGTCCGTatg (*phhA*) |
| PA1019 | *mucK* | cis.cis-muconate transporter | 3.2 | AG**AACAAGAATAat**g |
| PA1071-PA1073 | *braDEFG* | branched-chain amino acid transport proteins | 9.9 (*braD*), 3.2 (*braF*), 8.0 (*braE*), 4.3 (*braG*) | CCGG**AGG**TGG**AAAAAG**Tatg (*braE*) |
| PA1074 | *braC* | branched-chain amino acid transport protein | 3.2 | AC**AACAAT**GAC**AAC**ACAC**AAGAAGAGT**GG**AGC**ACTatg**AAGAAG** |
| PA1338 | *ggt* | gamma-glutamyltranspeptidase precursor | 11 | GAT**AACAATAAA**T**AGG**G**AAA**GCCCatg |
| PA1763 |  | hypothetical protein | 6.6 | **AAAAACAAGAACAAT**CTCTGGGGATAGCGCCatg |
| PA1891-PA1897 |  | hypothetical proteins | 13 (-91), 9.6 (-92), 18 (-93), 19 (-94), 19 (-95), 31 (-96), 36 (-97) | T**AAG**G**AAGAGGAGA**T**AAA**Tatg (PA1897) |
| PA1978 | *erbR* | glycerol metabolism activator | 22 | CAT**AAC**GAC**AATAAC**G**AGG**TGC**AGG**CCatg |
| PA1979-PA1980 | *eraSR* | two component regulator for quinoprotein ethanol dehydrogenase | 5.7 (*eraS*), 7.2 (*eraR*) |  |
| PA1981-PA1982 | *exaA* | quinoprotein ethanol dehydrogenase | 29 (-81), 58 (*exaA*) | atgAC**AACAAGAAC** (*exaA*) |
| PA1983-PA1984 | *exaBC* | ethanol utilization | 12 (*exaB*), 12 (*exaC*) | **AGGAAT**G**AAG**TGatg**AACAAGAACAAC** (*exaB*) |
| PA1991-PA1992 | *ercS* | ethanol utilization | 5.6 (-91), 5.1 (*ercS*) | **AACAATAAGAAT**GGTCTGTCAGatg (PA1991) |
| PA2003 | *bdhA* | 3-hydroxybutyrate dehydrogenase | 6.1 | **AGAAACAGC**atg |
| PA2006 |  | probable major facilitator superfamily (MFS) transporter | 13 | **AATAAGAAC**CTC**AGG**TACCTTGCGatg |
| PA2079 |  | probable amino acid permease | 6.0 | **AAGAAC**CCC**AATAAG**ATTCC**AGA**GGACATCC**AGa**tg**AAC** |
| PA2210 |  | probable MFS transporter | 21 | **AAGAACAACAATAAAAGA**GGTACGACCCatg |
| PA2247-PA2250 | *bkdA1A2B lpdV* | branched chain aminoacid assimilation | 3.2 (*bkdA1*), 6.2 (*bkdA2*), 6.0 (*bkdB*), 4.1 (*lpdV*) | C**AAC**GCC**AGGAGC**TCGGGGTatg (*bkdA2*) |
| PA2262 |  | putative 2-ketogluconate transporter | 35 | AC**AAGAACAAG**AC**AAG**CTC**AGGAGA**TACCCGACCatg |
| PA2264-PA2266 |  | gluconate dehydrogenase (PA2265) | 4.1 (-64), 3.8 (-65), 5.5 (-66) | **AGAAAG**ACG**AAA**CCGTGATGATCC**AGA**G**AGG**ACCCCACCatg (PA2264) |
| PA2507- PA2509 | *catBCA* | catechol degradation | 197 (*catB*), 87 (*catC*), 98 (*catA*) | GAC**AACAACAAG**C**AGA**GGCA**AAC**GCCatg (*catA*) C**AGA**CAG**AGGAGAAAC**GACCGatg (*catC*) |
| PA2511 | *antR* | transcriptional regulator | 11 | AA**AACAGA**GCGCC**AGCAGA**GCGCACCCCC**AGC**CTTGCCGTGTCG**AGT**GCCG**AAC**CatgATG**AGG** |
| PA2512-PA2514 | *antABC* | anthranilate degradation | 80 (*antA*), 67 (*antB*), 152 (*antC*) | CAT**AAC**GAC**AAC**GCAC**AAG**GGTG**AGA**ACGCatg (*antA*) |
| PA2515-PA2518 | *xylXYZL* | toluate degradation | 16 (*xylX*), 6.6 (*xylY*), 3.9 (*xylZ*), 7.2 (*xylL*) | G**AACAATAACAAC**GG**AGG**CCCCGGCCatg (*xylX*) |
| PA2519 | *xylS* | transcriptional regulator | 15 | AT**AACAACAAC**GAT**AGCAGC**CTG**AGT**GTCCTGCCCatg (*xylS*) |
| PA2760 | *oprQ* | outer membrane protein Q | 5.0 | **AACAAC**C**AGGAACAATAAG**atgTTG**AAGAAAAGG** |
| PA3186 | *oprB* | glucose/carbohydrate outer membrane porin | 5.7 | **AAa**tgTAC**AAGAACAAGAAA**ACC**AGA** |
| PA3366 | *amiEBCRS* | aliphatic amidase | 7.2 (*amiE*), 12 (*amiB*), 4.7 (*amiC*), 4.7 (*amiR*), 2.1 (*amiS*) | A**AAAAATAACAACAAGAGG**TGATATCCatg (*amiE*) C**AGAAGGAGT**TTCATCCatg (*amiB*) **AGC**GAC**AGT**CAC**AGGAGA**GG**AAA**CGGatg (*amiC*) **AACAATAACAAGAGG**GGTATCGTCatg (*amiS*) |
| PA3569-PA3570 | *mmsAB* | branched chain aminoacid assimilation | 5.0 (*mmsA*), 7.3 (*mmsB*) | **AACAATAAAAGC**CGGCCTGGCCGGTGC**AGGAGG**GTTCatg (*mmsA*) |
| PA3709 |  | probable MFS transporter | 30 | **AGA**C**AACAATAAGAGC**CCCCAC**AAG**GGGCGGG**AGA**GCCGC**Aat**g |
| PA4023-PA4025 | *eutPBC* | ethanolamine utilization | 57 (*eutP*), 4.5 (*eutB*), 2.1 (*eutC*) | GAT**AACAACAAG**ATTG**AGG**TG**AAAAAC**CCatg (*eutP*); |
| PA4091-PA4092 | *hpaAC* | hydroxyphenylacetate degradation | 16 (*hpaA*), 9.8 (*hpaC*) | **AGA**T**AACAACAAT**GACTGG**AGA**CGATGatg (*hpaA*) |
| PA4121-PA4123 | *hpcC* | hydroxyphenylacetate degradation | 29 (-21), 20 (-22), 90 (-23) | CC**AGGAGA**CACCACCAC**Aat**g (PA4121)  **AAT**G**AGGAAC**CGACCatg (PA4122) |
| PA4124-PA4125 | *hpcBD* | hydroxyphenylacetate degradation | 12 (*hpcB*), 17 (*hpcD*) |  |
| PA4126-PA4128 | *hpcG* | hydroxyphenylacetate degradation | 8.5 (-26), 11 (hpcC), 11 (-28) | CAC**AAAAAG**TAC**AAG**ATCatg (PA4126) |
| PA4137 |  | putative porin | 989 | **AAGAACAACAAC**C**AAG**GG**AAGAAT**CGatg |
| PA4209 | *phzM* | phenanzine specific methyltransferase | 37 | **AAC**G**AGA**GAG**AATAAAAGa**tg**AATAAT**TCG**AAT** |
| PA4496 |  | putative binding protein component of ABC transporter | 4.7 | **AGA**C**AAT**CAA**AATAAC**C**AGA**GGTCACTCCatg |
| PA4498 |  | putative metallopeptidase | 3.8 | **AGA**CG**AGG**AG**AACAACAAa**tg |
| PA4500 |  | putative ABC transporter protein | 19 | T**AGAAAAAGAAAAAAT**GAGGTTTGCatg |
| PA4501-PA4506 | *opdD* | glycine-glutamate dipeptide uptake | 77 (*opdD*), 28 (-02), 15 (-03), 20 (-04), 11 (-05), 11 (-06) | **AAC**GAC**AACAATAAA**GGG**AGCAAT**C**AGg**tg (*opdD*) |
| PA4588 | *gdhA* | glutamate dehydrogenase | 6.5 | C**AAAAAA**TTGTTCACAA**AAC**T**AGAAAC**CGGTGAG**AAAAat**g |
| PA4908-PA4912 |  | putative ABC transporter proteins | 8.0 (-08), 16 (-09), 24 (-10), 20 (-11), 53 (-12) | G**AAGAGA**CTG**AAAAat**g (PA4912) **AAGAGGAGC**TGTCatg (PA4909) |
| PA4913 |  | probable binding protein component of ABC transporter | 26 | A**AAC**AC**AACAACAAC**ATCGTAGTTACGAGCGCCAACATCATCCTC**AGGAGCAAC**ACCCCatg |
| PA4986 |  | probable oxidoreductase | 32 | **AACAACAAGAAG**GACGCCAACCgtg |
| PA5112 | *estA* | esterase | 2.8 | GAC**AATAAAAACAAA**TCATGG**AGTAAGAGA**atg |
| PA5152-PA5155 |  | putative permease of ABC transporter | 2.9 (-52), 5.0 (-53), 4.9 (-54), 3.2 (-55) | **AATAACAAGAGGAAA**ATCCatg (PA5152 first gene in operon) |
| PA5309 | *pauB4* | FAD-dependent oxidoreductase | 3.5 | **AATAAT**C**AGG**ATTTCACACatg |
| PA5380 | *gbdR* | Transcriptional regulator for glycine betaine catabolism | 7.4 | **AACAAT**CAT**AAAAAAAGC**CTCGTG**AGA**GGCCTGCTTGCTC**AGC**GTG**AGGAGA**TACACCCatg |
| PA5522-PA5523 | *pauA6* | glutamylpolyamine synthetase | 3.2 (-22), 4.3 (*pauA6*) | atgGCCCGCG**AGCAGCAAC** (PA5523) |
| PA5542 |  | hypothetical proteins | 7.6 | CAT**AAG**G**AAA**ACGTCCatg |
| PA5543-PA5545 |  | hypothetical proteins | 12 (-45), 30 (-44), 8.3 (-43) | GAC**AACAAC**GAT**AAGAACAGGAGA**CTTCCCatg (PA5545) |

^a^ Gene numbers and functions are taken from the *Pseudomonas* genome database [61]

^b^ data derived from Sonnleitner *et al* [31]

^c^ A-rich sequences are highlighted in bold; start codons are in lower case letters.
