# Supplementary material for: Regulation of Hfq by the RNA CrcZ in Pseudomonas aeruginosa Carbon Catabolite Repression
Source: PLoS Genet. 2014 Jun 19;10(6):e1004440. doi: 10.1371/journal.pgen.1004440 (PMC4063720; doi:10.1371/journal.pgen.1004440)
Supplement: Table S2 — Strains and plasmids used in this study. (DOCX) [file pgen.1004440.s012.docx]

**Table S2.**  Strains and plasmids used in this study

| **Strain/plasmid** | **Genotype/relevant features** | **Source/reference** |
| --- | --- | --- |
| ***P. aeruginosa*** |  |  |
| PAO1 |  | [62] |
| PAO1*hfq*- | *hfq::aadA*; Sp/Sm^r^ | [33] |
| PAOΔ*crc* | PAO6673 | [7] |
| PAO1*hfq*-Δ*crc* | *hfq::aadA,* Δ*crc;* Sp/Sm^r^ | This study |
| PAO1Δ*crcZ* | PAO6679 | [7] |
| PAO1*hfq*-Δ*crcZ* | *hfq::aadA,* Δ*crcZ;* Sp/Sm^r^ | This study |
|  |  |  |
| ***E. coli*** |  |  |
| XL1-Blue | *recA1 endA1 gyrA96 thi-1 hsdR17*(r_K_-, m_K_+) *supE44 relA1 lac* [F' *proAB* *lacI^q^lacZ*ΔM15::Tn*10*(Tc^r^)] | Stratagene |
| HB101 | *thi-1 hsdS20(rB-, mB-) supE44 recA13 ara-14 leuB6 proA2 lacY1 galK2 xyl-5 mtl-1 rpsL20* | [63] |
| AM111F’ | AM111 [*F‘ proAB lacI*^q^ *lacZ*ΔM15::Tn*10*; Tc^r^] | [64] |
| JW4130 | BW25113 *hfq*::Km^r^ | [65] |
|  |  |  |
| **Plasmids** |  |  |
| pUC19 | *colE1 ori* ; Ap^r^ | [66] |
| pET22b | T7 expression vector, encodes His-tag; Ap^r^ | Novagen |
| pRK2013 | Helper plasmid, ColE1 replicon, Tra; Km^r^ | [67] |
| pME6013 pME6014 pME6015 | Cloning vectors for translational *lacZ* fusions (pME6010 derivative); Tc^r^ | [68] |
| pME6016 | Cloning vector for transcriptional *lacZ* fusions; Tc^r^ | [68] |
| pMMB67HE | IncQ expression vector carrying an inducible P*_tac_* promoter; Ap/Cb^r^ | [69] |
| pME4510 | Broad–host-range promoter-probe plasmid, Gm^r^ | [70] |
| pME4510*hfq*_Flag_ | pME4510 carrying PAO1 *hfq* fused to a Flag-tag encoding sequence under control of its authentic promoter | This study |
| pME4510*hfq*_Y25D_ | pME4510*hfq*_Flag_ encoding the PAO1 Hfq_Y25DFlag_ protein | This study |
| pME4510*hfq*_K56A_ | pME4510*hfq*_Flag_ encoding the PAO1 Hfq_K56AFlag_ protein | This study |
| pMMBΔrbs | pMMB67HE with a deletion o the RBS of *lacZ* | This study |
| pMMB*crcZ* | pMMBΔrbs harboring *crcZ* under transcriptional control of P*_tac_* | This study |
| pMMB*hfq*_Flag_ | pMMB67HE with PAO1 *hfq* fused to the Flag-tag encoding sequence under transcriptional control of P*_tac_* and translational control of the T7 gene *10* RBS | This study |
| pMMB*crc*_Flag_ | pMMB67HE with *crc* fused to the Flag-tag encoding sequence under transcriptional control of P*_tac_* and translational control of the T7 gene *10* RBS | This study |
| pME3856 | pME6010 carrying *lacZ* under the control of P*_tac_* | [71] |
| pME9655 | pME6013 carrying a translational *amiE::lacZ* fusion | [7] |
| pME9658 | pME9655 with a 32-bp deletion of the *amiL* terminator (nt -65 to nt -33 with regard to the A (+1) of the *amiE* start codon) | This study |
| pME10011 | pME6015 carrying a translational *phzM::lacZ* fusion | [35] |
| pTL*hfq* | pME6014 carrying a translational *hfq::lacZ* fusion | This study |
| pTL*estA* | pME6015 carrying a translational *estA::lacZ* fusion | [36] |
| pTC*amiE* | pME6016 carrying the promoter of *amiE* fused to *lacZ* | This study |
| pME9672 | pME3087 with a 779-bp deletion of *crc* (nt +1 to nt +779 with regard to the A (+1) of the start codon) | [7] |
| pME9673 | pME3087 with a 160-bp deletion of the promoter of *crcZ* (nt -64 to nt +96 with regard to the transcriptional start site) | [7] |
| pME9659 | pET22b harbouring *crc* fused to the His-tag encoding sequence | [7] |
| pUBS520 | pET22b harbouring PAO1 *hfq* cloned into the *Nde*I/*Bam*HI site | [72] |
| pHfq_Pae_ | pUC19 derivative harbouring PAO1 *hfq* under transcriptional control of P*_lac_* and translational control of the T7 gene *10* RBS. | This study |
| pHfq_PaeY25D_ | pHfq_Pae_ encoding the PAO1 Hfq_Y25D_ mutant protein | This study |
| pHfq_PaeK56A_ | pHfq_Pae_ encoding the PAO1 Hfq_K56A_ mutant protein | This study |
