# Supplementary material for: Regulation of Hfq by the RNA CrcZ in Pseudomonas aeruginosa Carbon Catabolite Repression
Source: PLoS Genet. 2014 Jun 19;10(6):e1004440. doi: 10.1371/journal.pgen.1004440 (PMC4063720; doi:10.1371/journal.pgen.1004440)
Supplement: Table S3 — Oligonucleotides used in this study. (DOCX) [file pgen.1004440.s013.docx]

**Table S3.** Oligonucleotides used in this study

| **Name** | **Sequence**^a^ | **Restriction site** |
| --- | --- | --- |
| A1 | TTTTTT**GAATTC**GGCTGCATGCTATCTCAGGCGC | *Eco*RI |
| A2 | ACGT**GGATCC**GCCCATTTGCTCTGTACG | *Bam*HI |
| A5 | AA**TCTAGA**CGTAATACGACTCACTATAGATCAGGTCGTGCGC ATCAG | *Xba*I |
| A75 | TTTTT**GGATCC**tcacttgtcgtcatcgtctttgtagtcGGCCTCCTTCTCCAGT CC | *Bam*HI |
| B2 | ACGT**GGATCC**GTCCCGAAAAAATAACAAC | *Bam*HI |
| B5 | TTTT**CTGCAG**GGATAACAATTCCCCTCTAGAAAT | *Pst*I |
| C1 | TTTTTT**CTGCAG**GAAATATCGCCGTGACGCAT | PstI |
| C3 | GCACAACAACAATAACAAGCAACGACGAAG |  |
| C6 | ATGC**GGATCC**GAAATGGTGTAAGGCGAAGG | *Bam*HI |
| C78 | CCGCCACTCCGAC |  |
| C79 | TTTT**GGATCC**tcacttgtcgtcatcgtctttgtagtcAGCGTTGCCCGGCTCG | *Bam*HI |
| D79 | TTTT**GGATCCT**cacttgtcgtcatcgtctttgtagtcGATGCTCAACTGCCAGTC | *Bam*HI |
| D112 | GTACAGGGAACACGCAACC |  |
| E2 | CTTCTTCCGACTGGCTGCGGG |  |
| E6 | **TCTAGA**CGTAATACGACTCACTATAGGCACAACAACAATAA CAAGC | *Xba*I |
| E112 | CCCCGCCCACTCTTCAG |  |
| F70 | TTTTT**GAATTC**GGGGTGTCGATGAGCG | *Eco*RI |
| G70 | TTTT**CTGCAG**tcacttgtcgtcatcgtctttgtagtcAGCGTTGCCCGGCTCG | *Pst*I |
| G85 | GATAGTTAATGATCAGCCCACTG**ACGCGT**TG | *Mlu*I |
| H85 | TTTTTTT**AAGCTT**CCACACATTATACGAGCCGATG | *Hind*III |
| I26 | CCCCACACTACCATCGGCGATGCGTCG |  |
| I85 | TTTTTTT**AAGCTT**GCACAACAACAATAACAAGCAAC | *Hind*III |
| J85 | TTTTTTT**CTGCAG**AAAAACGGGGAAAACGGAGGGAGGG | *Pst*I |
| K3 | GCTGGAGTCGTTACGTGTTG |  |
| K74 | TTTTTT**CTGCAG**TGGCGCCACTTATAAGC | *Pst*I |
| L37 | ATCGTAGTCCGGATCGCAGT |  |
| M37 | AGTCATGAATCACTCCGTGGTA |  |
| M4 | CCAGCAGCAACGACACC |  |
| N4 | GCCACCGCGGTTTCC |  |
| N81 | GGTTTCCATCGATCTGGTCAAC |  |
| O81 | GTTGACCAGATCGATGGAAACC |  |
| P47 | GC**TCTAGA**AATATAATAGTTTAACTTT*AAGAAGGAGATATACA TA*TGTCAAAAGGGCATTCGCTACAAGACC | *Xba*I |
| P81 | GATGGTTTACGCGCACGCGATC |  |
| Q15 | TTTTTTTTTT**GGATCC**TCGGCGGGGTGTCG | *Bam*HI |
| Q47 | AA**GAATTC**CACCTGGGCGCTCAAAGAACAAAG | *Eco*RI |
| Q81 | GATCGCGTGCGCGTAAACCATC |  |
| Q99 | GCAGGCGCGGCATCTTGTAG |  |
| T27 | TTTTT**CTGCAG**GTAGCGAATGCCCTTTTGACAT | *Pst*I |
| W26 | CCG**TCTAGA**CGTAATACGACTCACTATAGCGTACAGGGAACA CG | *Xba*I |
| X26 | AAA**CTGCAG**GGCGACGAGTAAAACGGCAGGC | *Pst*I |

^a^ Restriction sites are highlighted in bold. The RBS of phage T7 gene *10* is in italics. The Flag-tag encoding sequence is depicted in lower case letters. The T7 promoter sequence is underlined.
