# Supplementary material for: Regulation of Hfq by the RNA CrcZ in Pseudomonas aeruginosa Carbon Catabolite Repression
Source: PLoS Genet. 2014 Jun 19;10(6):e1004440. doi: 10.1371/journal.pgen.1004440 (PMC4063720; doi:10.1371/journal.pgen.1004440)
Supplement: Text S1 — Supporting materials and methods and references. (DOCX) [file pgen.1004440.s014.docx]

**Supporting materials and methods**

Construction of plasmids

The plasmids and oligonucleotides used are listed in Tables S2 and S3, respectively.

**Plasmid pTC*amiE*:** To construct a transcriptional gene fusion between *amiE* and *lacZ*, a 109-bp fragment containing the *amiE* promoter region (nt -242 to nt -133 with regard to the A (+1) of the start codon) was amplified by PCR using the oligonucleotides A1/K74 and chromosomal DNA of PAO1 as template. The PCR fragment was cleaved with *Eco*RI and *Pst*I and then ligated into the corresponding sites of plasmid pME6016.

**Plasmid pME9658:** To render the *amiE::lacZ* fusion independent of AmiR-mediated anti-termination, the terminator upstream of the *amiE* gene was deleted. Two PCR fragments were generated using the primer pairs A1/A2 and B2/C1, respectively, which resulted in a 177-bp fragment located upstream of the *amiL* terminator (nt -242 to nt -65 with regard to the A (+1) of the *amiE* start codon) and a 53-bp downstream fragment (nt -33 to nt +20 with regard to the A (+1) of the *amiE* start codon). These two fragments were fused at their *Bam*HI sites and the resulting fragment was cloned into the *Eco*RI and *Pst*I sites of plasmid pME6013, resulting in plasmid pME9658.

**Plasmid pTL*hfq*:** To construct a translational *hfq::lacZ* gene fusion, a 585-bp fragment (nt -563 to nt +22 with regard to the A (+1) of the start codon) including the *hfq* promoter was amplified by PCR using the oligonucleotides Q15/T27 and chromosomal DNA of PAO1 as template. The PCR fragment was cleaved with *Bam*HI and *Pst*I and then ligated into the corresponding sites of plasmid pME6014, abutting the 7^th^ codon of *hfq* to the 8^th^ codon of *lacZ*.

**Plasmid pHfq_Pae_:** To construct a *hfq* over-expression plasmid for Hfq purification, a 283-bp fragment of *hfq* (nt +1 to nt +283 with regard to the A (+1) of the start codon) was amplified by PCR using the oligonucleotides P47/Q47 and chromosomal DNA of PAO1 as template. Oligonucleotide P47 contained a sequence derived from plasmid pET22b including the XbaI site and the RBS of phage T7 gene 10. The resulting PCR product was cleaved with XbaI and EcoRI and then ligated into the corresponding sites of plasmid pUC19. The resulting plasmid pHfq_Pae_ harbors *hfq* under transcriptional control of P*_lac_* (derived from pUC19) and translation is directed by the RBS of T7 gene *10*.

**Plasmid pHfq_PaeY25D_ and pHfq_PaeK56A_:** The Y25D mutation and the K56A mutation were generated by site directed mutagenesis using the QuickChange site-directed mutagenesis protocol ([Agilent](http://www.stratagene.com/manuals/200518.pdf) Technologies). The plasmid pHfq_Pae_ was used together with the mutagenic oligonucleotides N81/O81 (Y25D) and P81/Q81 (K56A), respectively. The entire plasmids were amplified with Pfu DNA polymerase (Thermo Scientific). The parental plasmid templates were digested with *Dpn*I and the mutated nicked circular strands were transformed into *E. coli* XL1-Blue, generating pHfq_PaeY25D_ and pHfq_PaeK56A_.

**Plasmids pMMB*hfq*_Flag_ and pMMB*crc*_Flag_:** The plasmids pUBS520 and pME9659 harboring the *hfq* and *crc* genes, respectively, were used as templates for PCR amplification together with oligonucleotides B5 (binds within the pET22b sequence) and C79 (*hfq*) and D79 (*crc*), respectively. C79 and D79 encode Flag-tag sequences. The PCR products encompass the *hfq* and *crc* genes abutted to the Flag-tag encoding sequence, respectively. The 303-bp *hfq*_flag_ fragment and the 834-bp *crc_flag_* fragment were cleaved with *Pst*I and *Bam*HI and then ligated into the corresponding sites of pMMB67HE, resulting in plasmid pMMB*hfq*_Flag_ and pMMB*crc*_Flag_, respectively.

**Plasmid pMMB*crcZ*:** The RBS of the *lacZ* gene was first deleted in plasmid pMMB67HE, which resulted in plasmid pMMB∆rbs. For this purpose, a 720-bp fragment was amplified by PCR using oligonucleotides G85 and H85 and pMMB67HE as template. G85 binds at position 719 to 689 nt upstream of the transcriptional start site of P*_tac_* and H85 introduced a *Hind*III site at the transcriptional start site of P*_tac_*. This fragment was cloned into the *Mlu*I and *Hind*III sites of plasmid pMMB67HE resulting in a 44-bp deletion of pMMB67HE including the RBS of the *lacZ* gene (nt +1 to nt +45 with regard to the transcriptional start site (+1) of P*_tac_*). Next, oligonucleotides I85 and J85 were used to amplify a 407-bp fragment of *crcZ* (nt +1 to nt +407 with regard to the transcriptional start site (+1) of *crcZ*) using chromosomal DNA as template. The PCR fragment was cloned into the *Hind*III-*Pst*I sites of plasmid pMMB∆rbs, resulting in plasmid pMMB*crcZ*.

**Plasmid pME4510*hfq*_Flag_:** To construct a *hfq_flag_* fusion gene under control of its authentic promoter, a 804-bp fragment of *hfq* (nt -558 to nt +246 with regard to the A (+1) of the start codon) was amplified by PCR using oligonucleotides F70/G70 and chromosomal DNA as template. The PCR fragment was cleaved with *Eco*RI and *Pst*I and then ligated into the corresponding sites of plasmid pME4510.

**Plasmids pME4510*hfq*_Y25D_ and pME4510*hfq*_K56A_:** The Y25D mutation and the K56A mutation were generated by site directed mutagenesis using the QuickChange site-directed mutagenesis protocol ([Agilent](http://www.stratagene.com/manuals/200518.pdf) Technologies). Plasmid pME4510*hfq*_Flag_ and the mutagenic oligonucleotides N81/O81 (Y25D) and P81/Q81 (K56A) were used to amplify the entire plasmid with Pfu DNA polymerase (Thermo Scientific). The parental plasmid templates were digested with *Dpn*I and the mutated nicked circular strands were transformed into *E. coli* XL1-Blue, generating plasmids pME4510*hfq*_Y25D_ and pME4510*hfq*_K56A_.

All DNA manipulations were verified by DNA sequencing.

CoIP and RT-PCR

PAO1*hfq*- harboring either the *hfq* over-expression plasmid pMMB*hfq*_Flag_ or the control plasmid pMMB67HE, grown in BSM medium supplemented with 40 mM succinate and 40 mM acetamide, were harvested (25 ml of culture) at an OD_600_ of 1.5. The medium contained 1 mM Isopropyl-β-D-thiogalactopyranosid (IPTG) to induce *hfq* expression. The cells were first resuspended in lysis buffer (20 mM Tris pH 8, 150 mM KCl, 1 mM MgCl_2_, 1 mM DTT) and then snap frozen in liquid nitrogen. The cells were lysed by sonication (6 times for 10 sec on ice) in 500 µl lysis buffer, containing 200 U RiboLock RNase inhibitor (Fermentas). Cell debris were removed by centrifugation and Hfq-specific antibodies (Pineda) were added to 400 µl supernatant and incubated for 2 hours at 4°C on a rotating wheel. Then, 20 µl Dynabeads Protein G beads (Novex) were added and the incubation was continued for 1 hour. The beads were washed three times with lysis buffer and total RNA was isolated by phenol/chloroform extraction. The RNA was treated with RNase free DNaseI (Roche) and resuspended in 10 µl of RNase free water. 3 µl were used for cDNA synthesis with AMV reverse Transcriptase (Promega) together with the oligonucleotides E2 (Table S3; binds to CrcZ), N4 (Table S3; binds to *amiE*) and E112 (Table S3; binds to RsmZ). 2 µl of cDNA derived from the co-immunoprecipitated RNA and from the unbound total RNA (isolated from the supernatant after CoIP) of PAO1*hfq*-(pMMB*hfq*_Flag_) and PAO1*hfq*-(pMMB67HE) (negative control) were used as a template for PCR amplification using the oligonucleotides M4 and N4 (Table S3; *amiE*), C3 and E2 (Table S3; CrcZ) or D112 and E112 (Table S3; RsmZ), respectively. The corresponding PCR products were 214 bp (*amiE*), 151 bp (CrcZ) and 96 bp (RsmZ) in length. Chromosomal DNA of PAO1 was used as a positive control. To confirm total DNA depletion of the RNA samples, the PCR reactions were performed with the same amount of RNA as used for the RT-PCR reaction without reverse transcriptase.

Filter binding assay

The *amiE*_-134-+20_ RNA (see Materials and Methods) was dephosphorylated with FastAP thermo sensitive alkaline phosphatase (Thermo Scientific) and subsequently 5´-end labeled using [γ-^32^P]-ATP (Hartmann Analytic) and polynucleotide kinase (Thermo Scientific). The labeled RNA was gel-purified and dissolved in diethylpyrocarbonate-treated water. Labeled RNA (10 nM) was incubated with increasing amounts of purified Hfq proteins in 10 mM Tris-HCl (pH 8.0), 10 mM MgCl_2_, 60 mM NaCl, 10 mM NaH_2_PO_4_, 10 mM DTT, and 75 ng tRNA in a total volume of 30 µl. Then, the samples were transferred to a Protran BA85 Nitrocellulose membrane (Whatman) (upper layer) and an Amersham Hybond-N membrane (GE Healthcare) (lower layer) by a Micro-Sample Filtration device (Schleicher & Schuell). The radioactively labeled bands were visualized with a PhosphorImager (Molecular Dynamics) and quantified with ImageQuant software 5.2.

Determination of *amiE* stability

The stability of *amiE* mRNA during CCR and in the absence of CCR was determined upon addition of rifampicin (100µg/ml final concentration) to PAO1 grown to an OD_600_ of 1.0 in BSM medium supplemented with either 40 mM acetamide (no CCR) or 40 mM acetamide and 40 mM succinate (CCR) and to PAO1*hfq*- grown to an OD_600_ of 1 in BSM medium supplemented with 40 mM acetamide and 40 mM succinate. Samples were withdrawn at several times thereafter (see Fig. S8) and total RNA was extracted by using the hot phenol method. 2 µg of total RNA was used for cDNA synthesis as described above with oligonucleotide N4 for *amiE* and M37 (Table S3) for 16S rRNA (internal control). 2 µl of cDNA were used as a template for PCR amplification using oligonucleotides M4 and N4 (Table S3) for amplification of *amiE* and L37 and M37 (Table S3) for amplification of 16S rRNA, respectively. The corresponding PCR products were 214 bp (*amiE*) and 192 bp (16S rRNA) in length. Chromosomal DNA of PAO1 was used as a positive control. To confirm total DNA depletion of the RNA samples, the PCR reactions were performed with the same amount of RNA as used for the RT-PCR reaction without reverse transcriptase (not shown).

Western-blot analyses

Equal amounts of total protein were separated on 12% SDS-polyacrylamide gels and then electro-blotted to a nitrocellulose membrane. The blots were blocked with 5% dry milk in TBS buffer, and then probed with rabbit anti-Hfq (Pineda), rabbit anti-Crc (Pineda) or with rabbit-anti S2 antibody directed against ribosomal protein S2 (loading control). The antibody-antigen complexes were visualized with alkaline-phosphatase conjugated secondary antibodies (Sigma) using the chromogenic substrates nitro blue tetrazolium chloride (NBT) and 5-Bromo-4-chloro-3-indolyl phosphate (BCIP).

Determination of the intracellular Hfq concentration

PAO1 was grown in BSM medium supplemented with 40 mM succinate to an OD_600_ of 2.0. The withdrawn samples were serially diluted and plated on LB plates to determine the colony forming units (CFU)/ml. To determine the Hfq concentration three samples were taken from three individual cultures (corresponding to 50µl culture of PAO1 at an OD_600_ of 2.0). The samples were centrifuged and resuspended in protein loading buffer. Either sample was separated on a 12% SDS-polyacrylamide gel together with 0.1, 0.4 and 0.6 pmol of purified Hfq-hexamer protein. The proteins were then electro-blotted to a nitrocellulose membrane. The blot was blocked with 5% dry milk in TBS buffer, followed by probing with rabbit anti-Hfq (Pineda) and anti-rabbit IR Dye 800 (Rockland). The antibody-antigen complexes were visualized with Odyssey CLx and Image Studio software (LiCor). The Hfq concentrations were determined with the aid of the defined amounts of Hfq protein loaded on the gel. The Hfq concentration(s) were then normalized to the CFU/ml and multiplied with the Avogadro constant resulting in the amount of Hfq-hexamer molecules/cell.

**Supporting References**

1. Winsor GL, Lam DK, Fleming L, Lo R, Whiteside MD, et al. (2011) *Pseudomonas* Genome Database: improved comparative analysis and population genomics capability for *Pseudomonas* genomes. Nucleic Acids Res 39: D596-600.
2. Holloway BW, Krishnapillai V, Morgan AF (1979) Chromosomal genetics of *Pseudomonas*. Microbiol Rev 43: 73-102.
3. Sambrook J, Russell DW (2001) Molecular Cloning: A Laboratory Manual. Cold Spring Harbour, New York: Cold Spring Harbor Press.
4. Sonnleitner E, Moll I, Bläsi U (2002) Functional replacement of the *Escherichia coli hfq* gene by the homologue of *Pseudomonas aeruginosa*. Microbiology 148: 883-891.
5. Baba T, Ara T, Hasegawa M, Takai Y, Okumura Y, et al. (2006) Construction of *Escherichia coli* K-12 in-frame, single-gene knockout mutants: the Keio collection. Mol Syst Biol 2: 2006-2008.
6. Yanisch-Perron C, Vieira J, Messing J (1985) Improved M13 cloning vectors and host strains: nucleotide sequence of M13mp18 and pUC19 vectors. Gene 33: 103-119.
7. Figurski DH, Helinski DR (1979) Replication of an origin-containing derivative of plasmid RK2 dependent on a plasmid function provided in trans. Proc Natl Acad Sci 76: 1648-1652.
8. Schnider-Keel U, Seematter A, Maurhofer M, Blumer C, Duffy B, et al. (2000) Autoinduction of 2,4-diacetylphloroglucinol biosynthesis in the biocontrol agent *Pseudomonas fluorescens* CHA0 and repression by the bacterial metabolites salicylate and pyoluteorin. J Bacteriol 182: 1215-1225.
9. Fürste JP, Pansegrau W, Frank R, Blöcker H, Scholz P, et al. (1986) Molecular cloning of the plasmid RP4 primase region in a multi-host-range *tacP* expression vector. Gene 48: 119-131.
10. Rist M, Kertesz MA (1998) Construction of improved plasmid vectors for promoter characterization in *Pseudomonas aeruginosa* and other Gram-negative bacteria. FEMS Microbiol Lett 169: 179–183.
11. Zuber S, Carruthers F, Keel C, Mattert A, Blumer C, et al. (2003) GacS sensor domains pertinent to the regulation of exoproducts formation and to the biocontrol potential of *Pseudomonas fluorescens* CHA0. MPMI 16: 634-644.
12. Nikulin A, Stolboushkina E, Perederina A, Vassilieva I, Bläsi U, et al. (2005) Structure of *Pseudomonas aeruginosa* Hfq protein. Acta Crystallogr D Biol Crystallogr 61: 141-146.
